# Involvement of ST6Gal I‐mediated α2,6 sialylation in myoblast proliferation and differentiation

**DOI:** 10.1002/2211-5463.12745

**Published:** 2019-12-10

**Authors:** Caroline Vergé, Amel Bouchatal, Frédéric Chirat, Yann Guérardel, Abderrahman Maftah, Jean‐Michel Petit

**Affiliations:** ^1^ PEIRENE EA 7500 Glycosylation and Cell Differentiation University of Limoges France; ^2^ UGSF UMR 8576 CNRS University of Lille Villeneuve d'Ascq France

**Keywords:** myoblastic fusion, *N*‐glycans, *Pax7*, sialylated glycoproteins, sialyltransferases

## Abstract

Myogenesis is a physiological process which involves the proliferation of myoblasts and their differentiation into multinucleated myotubes, which constitute the future muscle fibers. Commitment of myoblasts to differentiation is regulated by the balance between the myogenic factors Pax7 and MyoD. The formation of myotubes requires the presence of glycans, especially *N*‐glycans, on the cell surface. We examined here the involvement of α2,6 sialylation during murine myoblastic C2C12 cell differentiation by generating a *st6gal1*‐knockdown C2C12 cell line; these cells exhibit reduced proliferative potential and precocious differentiation due to the low expression of *Pax7*. The earlier fusion of *st6gal1*‐knockdown cells leads to a high fusion index and a drop in reserve cells (Pax7^+^/MyoD^−^). In *st6gal1*‐knockdown cells, the Notch pathway is inactivated; consequently, *Pax7* expression is virtually abolished, leading to impairment of the proliferation rate. All these results indicate that the decrease in α2,6 sialylation of *N*‐glycans favors the differentiation of most cells and provokes a significant loss of reserve cells.

## Graphical abstract

The knockdown of *st6gal1* in C2C12 murine myoblastic cells induces an increase in cell fusion and in myotube formation at the expense of cell proliferation and maintenance of a reserve cell pool. The decrease in α2,6 sialylated *N*‐glycans may affect the receptors that would be involved in the Notch signaling pathway that results in reduced expression level of Pax7.

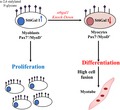

**Abbreviations**
EGF: epidermal growth factor
MAA: maackia amurensis agglutinin
MRF: myogenic regulatory factor
NICD: notch intracellular domain
PNGase F: *N*‐glycosidase F
Pofut1: protein *O*‐fucosyltransferase 1
Rbpj: recombination signal binding protein for immunoglobulin kappa J region
SNA: sambucus nigra amurensis

During vertebrate development, myogenesis begins with the formation and the migration of progenitor cells. Subsequently, their proliferation and fusion lead to the formation of myofibers. On the periphery of myofibers, satellite cells are considered as muscle stem cells since they can engage differentiation in response to an injury or during pathological processes 1, 2. When activated, satellite cells proliferate, then differentiate into myotubes, and mature into myofibers 3, 4. The fate of satellite cells is determined by the basic helix–loop–helix myogenic regulatory factors (MRFs) 5; these transcription factors play essential roles during postnatal growth, repair and regeneration of skeletal muscle 6. As hetero‐ or homodimers, they bind the E‐box (consensus sequence CANNTG) and activate the transcription of skeletal muscle‐specific genes 7. Satellite cells express other upstream transcription regulators belonging to the family of paired box proteins such as Pax3 and Pax7. *Pax7* holds a central position in satellite cell maintenance, since the knockdown of this gene results in the disappearance of satellite cells shortly after birth as well as in skeletal muscle atrophy 8, 9.

Myoblastic fusion depends on the glycosylation state of myoblasts. Among the glycogenes expressed in the murine myoblast cell line C2C12 and in satellite cells, several genes are transcriptionally deregulated during differentiation 10, 11. These results highlight the implication of glycans, and particularly of sialic acids in myoblast fusion and differentiation.

Sialic acids terminate glycan chains commonly found in cell surface glycoconjugates 12. Sialic acids play two main functions: (a) acting as biological masks, as some antirecognition agents 13; (b) being biological recognition sites as they are ligands for several molecules such as hormones or lectins 14. Glycan sialylation is under the control of sialyltransferases. At least twenty human sialyltransferases have been identified so far. They are classified into four groups according to the type of linkage and the nature of the acceptor: ST3Gal (ST3Gal I–VI), ST6GalNAc (I–VI), ST8Sia (I–VI), and ST6Gal (I and II) transferases 15. We focused on the latter group since ST6Gal I is the only α2,6 sialyltransferase expressed in human skeletal muscle 16.

Several signaling pathways have been shown to be implicated in the regulation of muscle cell differentiation. Among them, the Notch pathway and the *O*‐glycosylation of the receptor Notch epidermal growth factor (EGF)‐like repeats by the enzyme protein O‐fucosyltransferase 1 (Pofut1) are essential for the initiation of differentiation 17. Activation of the Notch signaling results in the release of notch intra cellular domain (NICD) into the cytosol, its translocation and binding to recombination signal binding protein for immunoglobulin kappa J region (Rbpj) in nucleus, and the subsequent transcription of downstream genes such as *Hes* and *Hey* 18. The products of these latter genes share the ability to prevent the heterodimerization of several MRF and notably *MyoD* 19, thus inhibiting myogenic differentiation.

In the present study, we created C2C12 clones in which the gene *st6gal1* encoding the ST6Gal I sialyltransferase is downregulated. We evidenced a reduced proliferative potential of *st6gal1‐*knockdown cells, due to a low expression of *Pax7*. These cells present an earlier differentiation and a decrease in the number of reserve cells. During differentiation of the control cells, we showed a global decrease in α2,6 sialylation, which concerns more particularly the *N*‐glycans. We concluded that α2,6 sialylation mediated by ST6Gal I is required for the activation of *Pax7*, limits the fusion of C2C12 myoblasts, and allows the upkeep of satellite cells.

## Methods

### Cell culture

C2C12 mouse myoblasts were grown to confluency under 5% CO_2_ at 37 °C in growth medium (GM) consisting of Dulbecco’s modified Eagle’s medium (DMEM) medium (Gibco, Carlsbad, CA, USA) supplemented with 10% (v/v) FBS (EuroBio, Courtaboeuf, France) and penicillin–streptomycin (100 µg·mL^−1^–100 units·mL^−1^; Gibco). To differentiate, cells were then switched to differentiation medium consisting of DMEM supplemented with 2% (v/v) horse serum (Gibco) and penicillin–streptomycin (100 µg·mL^−1^–100 units·mL^−1^) for different durations (from 24 to 336 h). Medium was routinely changed every 24 h.

When cells are confluent, they are trypsinized (0.125% trypsin and 0.125 mm EDTA) and harvested. At 120 h of differentiation, myotubes were isolated using a short trypsinization (0.1% trypsin and 0.1 mm EDTA; 30 s) that specifically left only reserve cells adherent to the flask 20.

### Proliferation assay

Cells were seeded at 72 000 cells per well in GM or GM with puromycin into six‐well plates. The cells were trypsinized (1% trypsin, 0.5 m EDTA; 5 min) and counted at each time point of the proliferation kinetics (0, 12, 24 and 48 h) with a Malassez chamber. Two replicates were analyzed at each time point, and an average of the values was calculated.

### Differentiation assay

At different stages of differentiation, cultured cells were washed twice with 1 mL PBS and fixed in 4% formaldehyde (Sigma‐Aldrich, Saint Louis, MO, USA) for 20 min at room temperature. Cells were then washed three times with 1 mL PBS before being dehydrated overnight at 4 °C in 70% (v/v) ethanol. Dehydrated cells were Jenner–Giemsa stained, and fusion index scoring was done as previously reported 21. Nuclei were counted in twelve randomly chosen microscope fields at a magnification of ×400. One microscope field usually contained between 100 and 200 nuclei. Fusion (%) was defined as [(number of nuclei in myotubes)/(number of total nuclei in myoblasts and myotubes)] × 100.

### Lentivirus production and st6gal1 knockdown in C2C12 cells

A mouse *st6gal1* shRNA lentiviral transfer vector was produced by annealing the primers presented in Table 1. A control shRNA was also created by annealing the primers sh‐mock‐UP and sh‐mock‐DN (Table 1). The annealed products were cloned into the *EcoRI* and *BamHI* sites of RNAi‐Ready pSIREN (BD Biosciences, Franklin Lakes, NJ, USA), and lentiviral particles were produced in HEK‐293T cells according to the manufacturer's instructions. After 48 h, the culture medium containing particles was recovered, filtered, and immediately used for C2C12 infection 22. C2C12 cells were incubated for 24 h with the retrovirus, and recombinant cells were selected in the presence of puromycin (Gibco) at a concentration of 10 µg·mL^−1^. The clonal populations were recovered and cultured separately in the same medium as C2C12 cells, except that puromycin was present at a final concentration of 2 µg·mL^−1^. Two clonal populations were selected and named C2C12‐sh‐Cl1 and C2C12‐sh‐Cl2. The same protocol was followed to create C2C12‐sh‐Mock cells.

**Table 1:** Sequences of the primers used to create the *st6gal1* shRNA vectors.

shRNA	Sequence (5′–3′)
sh1‐*st6gal1*‐UP	GATCCGCGGAACTATCTGAACATGAATAAAGTTCTCTTATTCATGTTCAGATAGTTCCTCTTTTTTACGCGTG
sh1‐*st6gal1*‐DN	AATTCACGCGTAAAAAAGAGGAACTATCTGAACATGAATAAGAGAACTTTATTCATGTTCAGATAGTTCCGCG
sh‐mock‐UP	GATCCGGGAATCTCATTCGATGCATACAAGTTCTCTGTATGCATCGAATGAGATTCTCTTTTTTACGCGTG
sh‐mock‐DN	AATTCACGCGTAAAAAAGAGAATCTCATTCGATGCATACAGAGAACTTGTATGCATCGAATGAGATTCCCG

### Release and purification of *N*‐glycans

Cells were resuspended in Triton X‐100 extraction buffer (1% Triton X‐100 in PBS buffer) and sonicated for 30 min. Debris and insoluble fraction were pelleted down by centrifugation at 18 900 ***g*** for 10 min at 4 °C; 0.1 m dithiothreitol was added to the supernatant (final concentration 10 mm), and the mixture was incubated at 37 °C for 1 h; addition of 0.5 m iodoacetamide (final concentration 50 mm) was followed by 1‐h incubation in the dark at 37 °C. The reduced/alkyled glycoproteins were precipitated with 1/9 volume of 100% trichloroacetic acid at −20 °C for 30 min. The pellet obtained by centrifugation at 18 900 ***g*** for 10 min at 4 °C was resuspended and washed with 1 mL of cold acetone and then centrifuged at 18 900 ***g*** for 10 min at 4 °C; this step was repeated three times. Sample was incubated overnight at 37 °C with trypsin (Sigma‐Aldrich) in 50 mm NH_4_HCO_3_, pH 8.4. The reaction was stopped by boiling at 100 °C for 5 min. *N*‐glycans were released by *N‐*glycosidase F (PNGase F; BioLabs, Ipswich, MA, USA) digestion at 37 °C for 1 day; *N‐*glycans and *O‐*glycopeptides were separated by C_18_ Sep‐Pak Chromatography (Waters, Milford, MA, USA). C_18_ Sep‐Pak was activated in pure acetonitrile (ACN), equilibrated in 5% aqueous acetic. Sample was loaded on the C_18_ Sep‐Pak, and the released *N*‐glycans were eluted with 5% aqueous acetic acid.

### Mass spectrometry analysis of glycans

Glycans were permethylated according to the method of Ciucanu and Kerek prior to mass spectrometry analysis 23. Briefly, samples were incubated with DMSO/NaOH/ICH_3_ during 2 h under agitation. The derivatization was stopped by addition of water, and the permethylated glycans were extracted in CHCl_3_ and washed at least eight times with water. Permethylated glycans were solubilized in ACN and mixed with 2.5‐dihydroxybenzoic acid matrix solution [10 mg·mL^−1^ dissolved in ACN/H_2_O (7 : 3, v/v)] and spotted on MALDI plate. MALDI‐TOF mass spectra were acquired on Shimadzu, AXIMA Resonance MALDI‐QIT‐TOF‐MS (Shimadzu, Kyoto, Japan).

### Quantification of *N*‐glycans

Quantification was carried out by adding voltage values (in mV) of the three major isotopes of each glycan structure. For each sample, percentages of individual glycan were then calculated.

### Quantitative real‐time RT‐PCR

RNA was isolated from cells with the RNeasy Mini Kit (Qiagen Inc, Hilden, Germany). RNA amounts were measured using a NanoDrop 1000 spectrophotometer (NanoDrop Technologies, Wilmington, DE, USA). cDNA was synthesized from 2 µg of total RNA using the High‐Capacity cDNA Reverse Transcription Kit (Applied Biosystems, Foster City, CA, USA). Levels of transcripts for specific genes were quantified in triplicate using 20 ng of cDNA for each sample, and TaqMan qRT‐PCR with specific probes and primers (Applied Biosystems) for *st6gal1* (Mm00486119_m1)*, Pax7* (Mm03053796_m1), *Hes1* (Mm00468601_m1), *Hey1* (Mm00468865_m1), *Hes6* (Mm00517097_g1), and *Rbpj* (Mm00770450_m1). *Gapdh* (Mm99999915_m1) was used as a reference gene. All probes and primers were purchased from Applied Biosystems.

Fluorescence was monitored on the QuantStudio 3 Real‐Time PCR Systems (Applied Biosystems) and quantified by the QuantStudio™ Design and Analysis Software v1.3 (Applied Biosystems). The comparative threshold cycle (*C*_t_) method (ΔΔ*C*_t_) was used to quantify the relative abundance of each mRNA 24. Comparative gene expression ΔΔ*C*_t_ represents the Δ*C*_t_ for each gene in a given condition (C2C12‐sh‐Cl2 cells) minus Δ*C*_t_ value of the same gene in the exogenous control condition (C2C12‐sh‐Mock) serving as a calibrator. Relative quantification of the transcripts in a sample reflects expression changes in the sample of interest compared to the calibrator sample, after normalization with *gapdh* as reference.

### Immunofluorescent staining

Cells were seeded into a 4‐well Lab‐Tek II chamber slide (Sigma‐Aldrich). After 24 h, cells were washed three times in 1 mL 1× PBS and fixed with 4% PFA‐PBS for 20 min. Cells were treated with PNGase F (1 : 600; Roche) for 1.5 h at 37 °C under 5% of CO_2_. Untreated cells were incubated in PBS for 1.5 h in the same culture conditions. Cells were washed thrice in 1× PBS, permeabilized with HEPES–Triton buffer [20 mm HEPES, 300 mm sucrose, 50 mm NaCl, 3 mm MgCl_2_, 0.5% (v/v) Triton X‐100, pH 7.4] for 30 min at 4 °C, then three 5‐min washes in 1× PBS were done. Cells were blocked for 1 h at room temperature in blocking buffer consisting of 10% goat serum, 1% BSA, and 0.1% Triton X‐100 in 1× PBS solution. Cells were incubated with a primary antibody overnight at 4 °C and then washed three times with 1 mL of 1× PBS‐0.1% Tween‐20 solution. An appropriate secondary antibody was then added, and incubation was continued for 15 min in the dark at 37 °C in a wet environment. Cells were then stained with DAPI (1 : 1000; Thermo Fisher, Waltham, MA, USA) and finally washed three times with 1 mL of 1X PBS–0.1% Tween‐20 solution. A slice was mounted with Mowiol solution, and the images were acquired with a LEICA inverted epifluorescence microscope (DMI 6000B) using identical exposure settings with the metamorph software (Molecular Devices, Sunnyvale, CA, USA). Individual images were taken and assembled with MetaMorph software. Primary antibodies used were against: Pax7 (100 µg·mL^−1^, Developmental Studies Hybridoma Bank, University of Iowa) and MyoD C‐20 (1 : 1000; Santa Cruz Biotechnology, Dallas, TX, USA). Secondary antibodies were goat anti‐mouse Alexa Fluor^®^ 488 (1 : 1000; Thermo Fisher Scientific) and goat anti‐rabbit Alexa Fluor^®^ 546 (1 : 1000; Thermo Fisher Scientific) for Pax7 and MyoD, respectively.

### Lectin labeling

A fluorescent labeling with maackia amurensis agglutinin (MAA) and sambucus nigra amurensis (SNA) lectins was also performed as described above except that the digoxigenin‐labeled lectin was used at 50 µg·mL^−1^ (for MAA) and at 5 µg·mL^−1^ (for SNA) and that it was detected with DyLight 488 Anti‐DIG (green for MAA; 1 : 1000; Vector Laboratories, Burlingame, CA, USA) and Streptavidin Alexa Fluor 568 conjugate (red for SNA; 1 : 200; Invitrogen, Carlsbad, CA, USA).

### Protein preparation and western blotting

Total cell extracts were prepared by solubilizing cell pellets in a radioimmunoprecipitation assay (RIPA) lysis buffer [50 mm Tris/HCl, pH 8, 50 mm NaCl, 1% (v/v) Triton X‐100, 0.5% (w/v) sodium deoxycholate, 0.1% SDS] and a cocktail of protease and phosphatase inhibitors (Roche). Cells were then centrifuged (12 000 ***g*** for 30 min at 4 °C). Protein concentration was determined using the bicinchoninic acid assay procedure (Thermo Scientific, Rockford, IL, USA). Equal amounts of proteins (100 µg) were resolved on a 10% polyacrylamide gel under denaturing and reducing conditions (migration at 20 mA for 1.5 h). The proteins were transferred onto a nitrocellulose blotting membrane (Premium 0.2 µm NC; GE Healthcare Life Sciences, Chicago, IL, USA) at 48 mA for 1.5 h. Then, membranes were blocked using 5% nonfat dry milk or BSA (Sigma‐Aldrich; w/v) in TBST (50 mm Tris/HCl, 150 mm NaCl, 0.1% Tween‐20) for 1 h at room temperature, followed by incubation overnight at 4 °C with specific primary antibodies diluted in 2.5% nonfat dried milk or BSA (w/v) in TBST. The following antibodies were used for immunoblotting: Pax7 (1 : 100; Developmental Studies Hybridoma Bank, University of Iowa), NICD (1 : 500; Cell Signaling Technology, Danvers, MA, USA), and gapdh (1 : 1000; R&D Systems, Minneapolis, MN, USA). Incubation in primary antibodies was followed by three washes of 5 min each in 0.1% TBST. Blots were incubated with horseradish peroxidase (HRP)‐coupled secondary antibody in 2.5% nonfat dry milk or BSA in 0.1% TBST for 1 h at room temperature. After three additional washes in TBST, reactive proteins were visualized with the BM Chemiluminescence Blotting Substrate (POD; Roche Applied Science) and the Amersham Imager 600 chemiluminescence detection camera (GE Healthcare Life Sciences). Band intensities were measured using the aforesaid system and its software. All bands were normalized to the corresponding gapdh band intensity.

### Glycoprotein analysis using lectin blot

Membrane glycoproteins were detected by lectin blot. Proteins were extracted with a RIPA buffer and were treated with PNGase F (Enzymatic Protein Deglycosylation Kit; Sigma‐Aldrich). An electrophoresis analysis was performed as explained below except that only 25 µg of proteins was loaded. After transfer on nitrocellulose blotting membrane (Premium 0.2 µm NC; GE Healthcare Life Sciences) at 130 mA for 1.5 h, a treatment was applied with PBS supplemented with 10% (v/v) blocking reagent (Roche) for 30 min at room temperature. After three washings with TBS and lectin buffer 1 for MAA lectin or with TBS (for SNA binding), membranes were incubated with lectins MAA (125 µg·mL^−1^, DIG glycan differentiation kit; Roche) and SNA (5 µg·mL^−1^; Vector Laboratories). Then, we performed three washes of 10 min in TBS for MAA and PBS‐0.05% Tween‐20 for SNA. We incubated the MAA membrane in α‐digoxigenin‐alkaline phosphatase or SNA membrane in streptavidin‐HRP during 1 h. We finished by three 10‐min washes in TBS 1X for MAA and PBS‐0.05% Tween‐20 solution for SNA.

Two control glycoproteins were also used: carboxypeptidase Y (an asialoprotein) as a negative control and fetuin (a sialylated protein) as a positive control.

## Results

### A lower expression of st6gal1 leads to an increase in the fusion rate of cells

Because the α2,3 linkage of sialic acids depends on six different α2,3 sialyltransferases (ST3Gal I–VI), and α2,6‐linked sialic acids are added by only two α2,6 sialyltransferases (ST6Gal I and II), we chose to work on α2,6 sialylation. Since ST6Gal II is not expressed in C2C12 cells, we focused our study on the α2,6 linkage of sialic acids by ST6Gal I.

**Figure 1:**
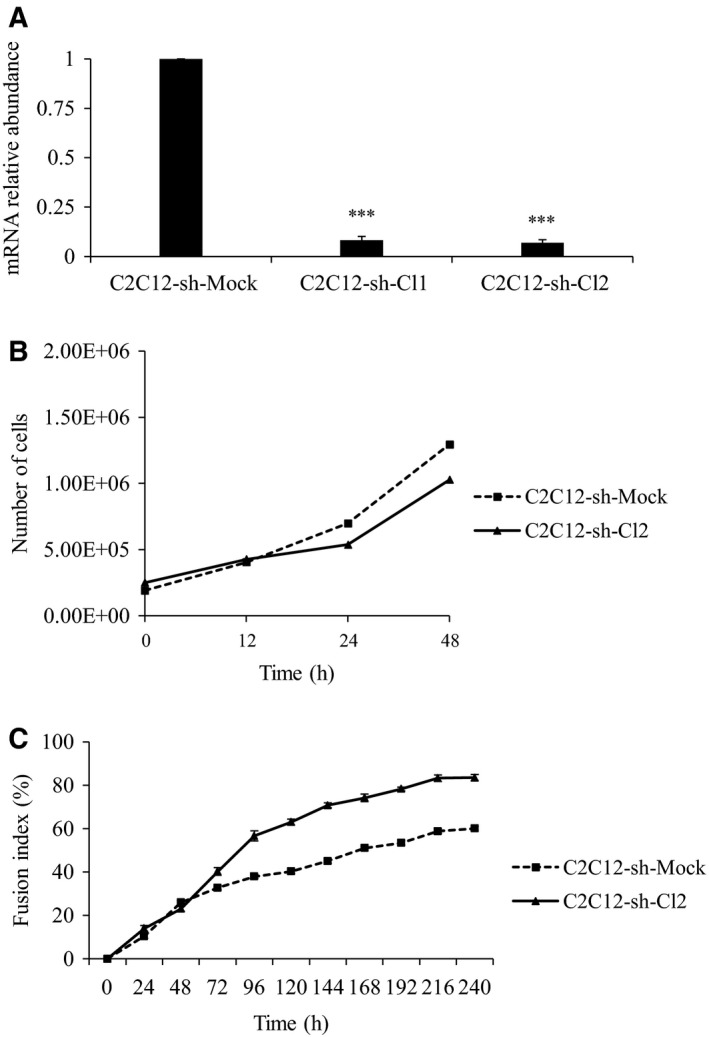
*st6gal1* knockdown modifies the proliferation rate and the differentiation rate. (A) Gene expression of *st6gal1* in C2C12 cells infected with mock shRNA (C2C12‐sh‐Mock) and C2C12 cells infected with *st6gal1*‐shRNA (C2C12‐sh‐Cl1, C2C12‐sh‐Cl2). *st6gal1* expression in C2C12‐sh‐Mock was used as reference. Data are expressed as relative transcript amounts corresponding to the ratio of *C*_t_ values for sh‐treated cells and *C*_t_ values for C2C12‐sh‐Mock cells and normalized against the expression of *gapdh* transcripts. Experiments have been done three times (*n* = 3). Means ± SEMs are shown (two‐tailed *t*‐test, with a significant level of ***P < 0.001). (B) Proliferation rate of C2C12‐sh‐Mock cells (dotted line) and C2C12‐sh‐Cl2 cells (solid line). Two different sets of measures are shown (*n* = 2), and the average curve is represented. (C) Differentiation rate of C2C12‐sh‐Mock (dotted line) and C2C12‐sh‐Cl2 cells (solid line). Cell fusion was measured at various times by Jenner–Giemsa staining and expressed as fusion index (%). Vertical bars denote SEM for twelve observation fields of the same experience (*n* = 1).

We generated C2C12 clones which expressed shRNA targeting the *st6gal1* transcript and measured the expression level of *st6gal1* by quantitative RT‐PCR (Fig. 1A). Compared with mock cells, the level of *st6gal1* mRNA decreased at least by a factor 12 in clones 1 and 2. The trends observed during the proliferation time course and the fusion index were almost the same for the two clones (Fig. S1). This confirms the cellular phenotype associated with *st6gal1* knockdown. We retained for further studies the clone 2. The proliferation time course has been carried out for 48 h (Fig. 1B). We observed a significant 25% decrease in cell proliferative capacity of clone 2 compared to mock cells. The fusion index followed until 240 h (Fig. 1C) revealed a more important fusion for *st6gal1*‐knockdown cells (C2C12‐sh‐Cl2 cells). During the first 72 h of differentiation, fusion rates of *st6gal1*‐knockdown cells and mock cells were quite similar, then after, the fusion rate of control cells slowed down, contrary to *st6gal1*‐knockdown cells. At 96 h of differentiation, fusion indices were about 38% for control cells and about 50% for *st6gal1*‐knockdown cells. At 240 h, it reached ~ 60% for mock cells and 80% for *st6gal1*‐knockdown cells.

### Knockdown of st6gal1 induces a drop in α2,6‐linked structures attached to glycans

To confirm the decrease in α2,6 sialylation during the differentiation of *st6gal1*‐knockdown cells, we have performed immunocytochemistry and lectin‐blot analyses during proliferation and differentiation of C2C12‐sh‐Mock and C2C12‐sh‐Cl2 cells.

**Figure 2:**
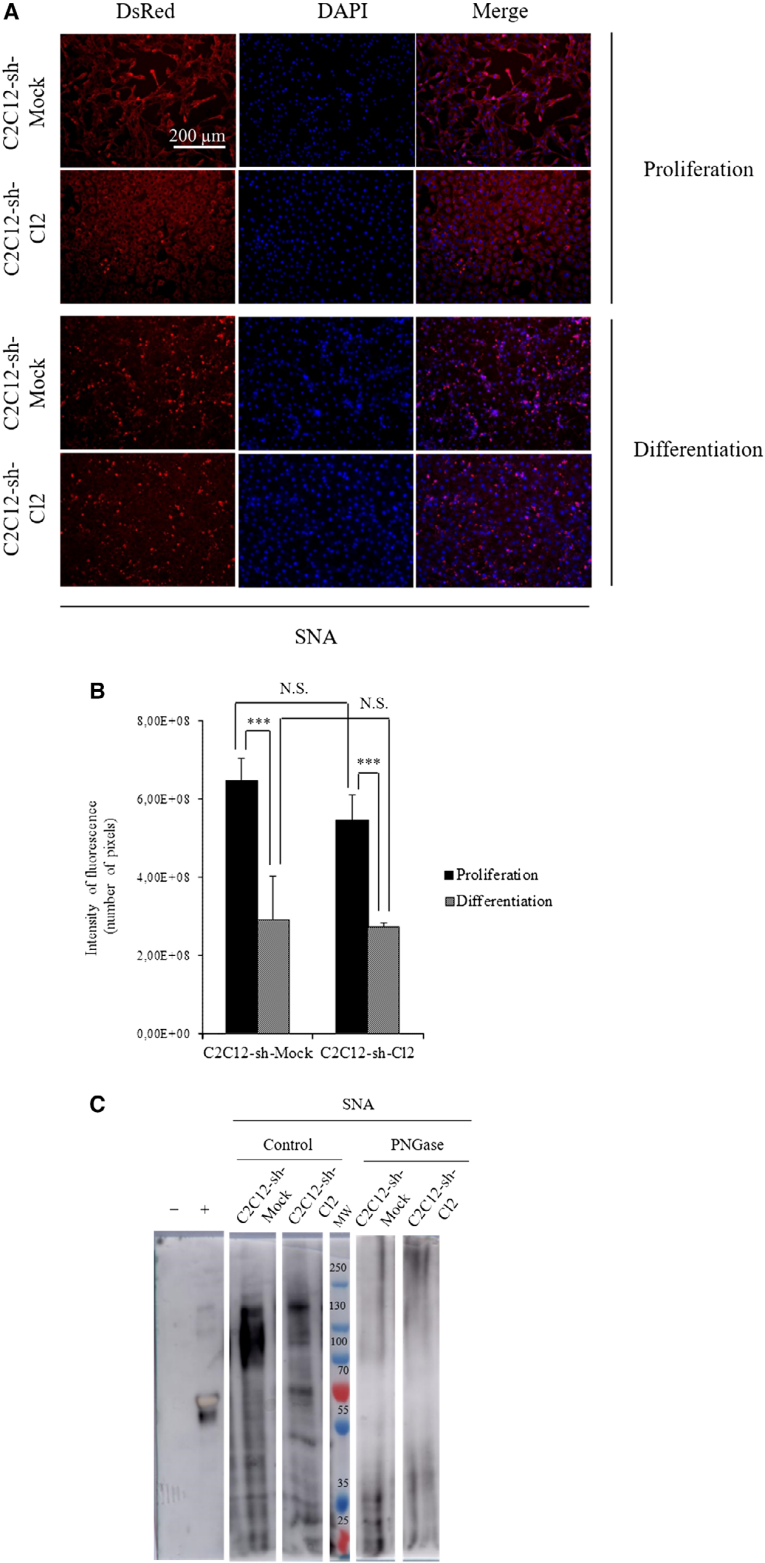
Knockdown of *st6gal1* reduced the α2,6 sialylation at the cell surface. (A) Immunocytochemistry of α2,6 sialylation in proliferating or differentiating cells. Photographs are organized from left to right: lectin labeling for proliferation or differentiation cells with lectin coupled to DsRed, nuclear staining with DAPI, and merge (lectin and DAPI stainings). (scale bar: 200 µm). (B) Quantifications of fluorescence intensities of C2C12‐sh‐Mock and C2C12‐sh‐Cl2. The fluorescence levels are measured with the software imagej (NIH, Bethesda, MD, USA) on three different fields. Means ± SEMs are shown (two‐tailed *t*‐test, with a significant level of ***P < 0.001). (C) Lectin‐blot analysis of C2C12‐sh‐Mock and C2C12‐sh‐Cl2 in proliferation treated or not with PNGase F using SNA. The negative control is carboxypeptidase Y (molecular weight: 63 kDa), and the positive control is fetuin (molecular weight: 68 kDa). The different bands and the controls come from the same blot, but some bands have been cut to conserve only the interesting ones. Each experiment has been performed three times (*n* = 3).

We looked for terminal α2,6‐linked sialic acids with DsRed‐SNA (Fig. 2A), and we observed, during cell proliferation, a decrease of around 16% in SNA binding in C2C12‐sh‐Cl2 cells compared with C2C12‐sh‐Mock cells. During cell differentiation, SNA binding decreased in both conditions; however, its level remained slightly lower in C2C12‐sh‐Cl2 cells compared with C2C12‐sh‐Mock cells (Fig. 2B). These two results were confirmed by lectin‐blot analyses of membrane glycoproteins of proliferating cells (Fig. 2C). Mainly glycoproteins of molecular weight higher than 55 kDa seem to possess α2,6 sialylated *N*‐glycans. We observed a decrease in the α2,6‐linked sialic acids in C2C12‐sh‐Cl2 cells compared with C2C12‐sh‐Mock cells. In order to check the specificity of SNA binding, PNGase F was used to remove *N*‐glycans. This treatment greatly reduced the staining of all the glycoproteins (Fig. 2C).

**Figure 3:**
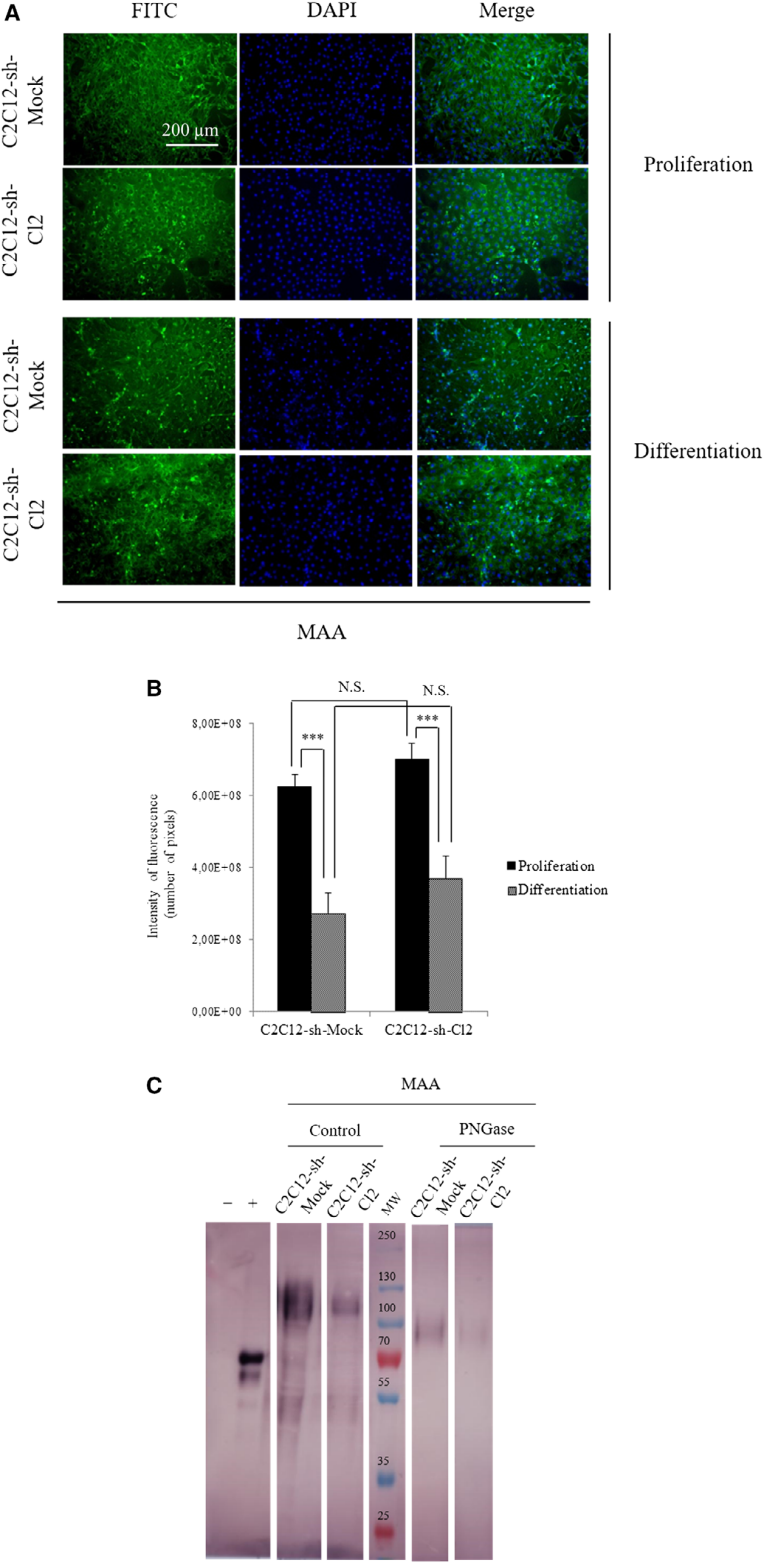
Knockdown of *st6gal1* did not modify significatively the α2,3 sialylation at cell surface. (A) Immunocytochemistry of α2,3 sialylation in proliferating or differentiating cells. Photographs are organized from left to right: lectin labeling for proliferation or differentiation cells with lectin coupled to FITC, nuclear staining with DAPI, and merge (lectin and DAPI stainings). (scale bar: 200 µm). (B) Quantification of fluorescence intensities of C2C12‐sh‐Mock and C2C12‐sh‐Cl2. The fluorescence levels are measured with the software imagej on three different fields. Means ± SEMs are shown (two‐tailed *t*‐test, with a significant level of ***P < 0.001). (C) Lectin‐blot analysis of C2C12‐sh‐Mock and C2C12‐sh‐Cl2 in proliferation treated or not with PNGase F using MAA. The negative control is carboxypeptidase Y (molecular weight: 63 kDa), and the positive control is fetuin (molecular weight: 68 kDa). The different bands and the controls come from the same blot, but some bands have been cut to conserve only the interesting ones. Each experiment has been performed three times (*n* = 3).

Then, we evaluated the α2,3‐linked sialic acids with FITC‐MAA (Fig. 3A). The staining was less intense during differentiation than during proliferation in clone 2 compared with control. FITC‐MAA staining is 10% stronger in C2C12‐sh‐Cl2 cells compared to C2C12‐sh‐Mock control cells during myoblast proliferation and differentiation (Fig. 3B). According to the lectin‐blot results (Fig. 3C), before PNGase F treatment, MAA staining was slightly lower in C2C12‐sh‐Cl2 cells compared with control cells, which is different from the lectin labeling presented in Fig. 3A. This discrepancy could be due to the fact that glycoproteins and glycolipids are detected in whole cells while lectin‐blot analyses only detect membrane glycoproteins. For the two cell populations, the labeling was more intense with high molecular weight glycoproteins (> 55 kDa). After PNGase F treatment, MAA staining decreased in a comparable manner for the two cell types. We conclude that the inhibition of *st6gal1* in C2C12 cells induced a strong drop in α2,6 sialylation of *N*‐glycans borne by high molecular weight glycoproteins (> 55 kDa).

To confirm the type of *N*‐glycans on which sialic acids are preferentially linked, C2C12‐sh‐Mock and C2C12‐sh‐Cl2 cells were treated with PNGase F in order to remove *N*‐glycans. Treated C2C12‐sh‐Mock cells present a weaker labeling of α2,6‐linked sialic acids compared with untreated mock cells (Fig. S2A). This observation confirms that α2,6‐linked sialic acids are preferentially linked to *N*‐glycans. Difference in α2,3‐linked sialic acid staining is less important after PNGase treatment whatever the cell population (Fig. S2B). This result agrees with the lectin‐blot analysis of PNGase‐treated cells (Fig. 3). This may be due to the presence of large amounts of sialylated glycolipids and of *O*‐glycans as well.

### Analysis of *N*‐glycans borne by glycoproteins during myoblastic differentiation

**Figure 4:**
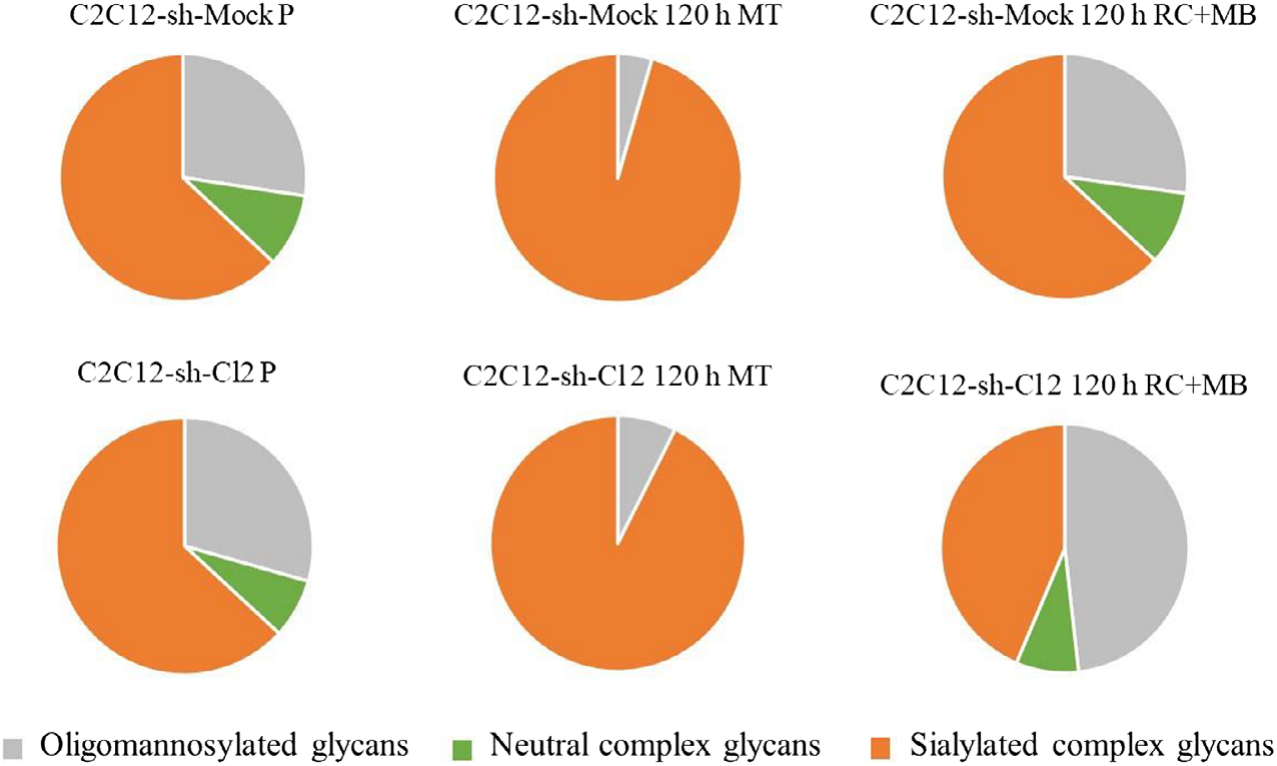
Distribution of sialylated and/or fucosylated *N*‐glycans in C2C12‐sh‐Mock and C2C12‐sh‐Cl2 cells. Distribution of the sialylated and nonsialylated *N*‐glycans compared to the total *N*‐glycans in proliferating myoblasts (P), in myotubes (MT), and in a mix of reserve cells (RC) and myoblasts (MB) at 120 h for C2C12‐sh‐Mock and C2C12‐sh‐Cl2. Mass spectrometry experiment has been done one time (*n* = 1).

We have performed mass spectrometry analyses of *N*‐glycans in C2C12‐sh‐Mock and C2C12‐sh‐Cl2 cells during proliferation and after 120 h of differentiation. In these experiments, myotubes were isolated following a short trypsinization after which reserve cells and myoblasts were collected. Twenty *N*‐glycans were identified according to their molecular mass (Fig. S3) and classified according to their nature (oligomannoses and complex glycans; see Fig. S4). *N*‐glycans were extracted from equal numbers C2C12‐sh‐Cl2 and C2C12‐sh‐Mock cells, and their respective amounts were evaluated (Fig. S4). During proliferation, there was no change in the percentage of sialylated glycans in both C2C12‐sh‐Cl2 and C2C12‐sh‐Mock cells (Fig. 4, Table 2).

**Table 2:** Classification of sialylated and/or fucosylated *N*‐glycans identified by MS/MS spectrometry. The identified glycans are classified according to their structure (oligomannosylated, neutral complex or sialylated complex glycans) and to their sialylated level (mono‐, bi‐, tri‐ or tetra‐sialylated). Mass spectrometry experiment has been done one time (*n* = 1). Cells of three independent cultures have been pooled before *N*‐glycans extraction and analyses.

		Mass (*m*/*z*)	C2C12‐sh‐Mock			C2C12‐sh‐Cl2		
			Proliferation	Differentiation		Proliferation	Differentiation	
				120 h MT	120 h RC + MB		120 h MT	120 h RC + MB
Oligomannosylated glycans		1579	9.89	1.50	7.16	7.63	2.41	9.52
		1783	6.37	1.46	7.53	9.40	3.05	16.31
		1988	3.83	0.47	3.58	4.39	0.87	6.74
		2192	3.74	0.55	4.44	4.76	1.09	8.19
		2395	3.57	0.49	4.47	3.22	0.00	7.46
		Total	27.40	4.47	27.18	29.41	7.43	48.22
Neutral complex glycans		2519	3.08	0.00	2.79	3.45	0.00	3.67
		2693	3.11	0.00	3.54	4.01	0.00	4.48
		2839	3.40	0.00	3.38	0.00	0.00	0.00
		Total	9.59	0.00	9.71	7.46	0.00	8.15
Sialylated complex glycans	Mono‐sialylated	2605	4.98	4.11	5.25	4.04	6.45	7.64
		3053	0.00	0.00	1.02	0.00	0.00	1.17
		2778	6.87	11.64	6.26	5.94	11.06	3.72
		2822	3.48	8.48	2.77	3.54	7.66	0.00
		3023	0.00	2.56	1.91	0.00	0.00	0.00
		3228	2.02	3.48	5.84	1.73	2.89	1.89
		Total	17.35	30.26	23.04	15.26	28.06	14.42
	Bi‐sialylated	2792	13.70	30.91	11.92	13.40	23.52	10.07
		3037	0.00	2.80	0.00	1.71	0.00	0.00
		3242	4.09	8.56	2.98	4.44	5.74	2.08
		2966	2.53	2.06	2.55	2.29	3.50	4.68
		Total	20.31	44.33	17.45	21.84	32.76	16.83
	Tri‐sia	3604	20.14	16.79	16.14	21.43	26.13	9.60
	Tetra‐sia	3966	5.20	4.15	6.47	4.59	5.62	2.78
	Total		63.01	95.53	63.11	63.13	92.57	43.62

We have focused on the percentage of each type of sialylated and nonsialylated *N*‐glycans among the total *N*‐glycans during myogenic differentiation (Fig. 4, Table 2, Fig. S5). The study of the percentages showed that the glycans (*m*/*z*: 2792, 3037, 3242, 2822, 2778, 3023, 3228) were more present in control than in clone 2 (1.4‐fold more in Mock cells in comparison with clone 2; Fig. 4, Table 2); the percentage of the biantennary bisialylated glycan (*m*/*z*: 2792) in C2C12‐sh‐Mock myotubes was 1.3 times higher than in C2C12‐sh‐Cl2 myotubes.

At 120 h of differentiation, the percentage of sialylated complex *N*‐glycans in the reserve cells (a mix of reserve cells and myoblasts) of clone 2 was 1.5 times less than in the reserve cells of the Mock (Fig. 4, Table 2). The most affected *N*‐glycans were the tri‐ and tetrasialylated *N*‐glycans (1.58‐ and 2.19‐fold drops, respectively, between clone 2 and mock reserve cells), in contrast to oligomannoses, which increased 1.83‐fold (Fig. 4, Table 2).

In addition, during this study, we have also compared the distribution of glycans in proliferative and reserve cells in clone 2 and in control cells. Proliferative and differentiated C2C12‐sh‐Mock cells do not present any significant difference in their respective distribution of glycans (Fig. 4, Table 2) except that abundance of three triantennary structures significantly evolved during cell differentiation, that is: *m*/*z* 3604 presented a 1.3‐fold drop, while *m*/*z* 3023 and 3228 showed a 2.7‐fold increase. In contrast, differentiation of clone 2 led to a marked increase of oligomannoses (1.8‐fold) and to a decrease of the sialylated glycans (1.4‐fold) in the population containing reserve cells than in C2C12‐sh‐Mock cells whereas for the last they represented about the same percentage of total *N*‐glycans (Fig. 4, Table 2).

### A strong drop in Pax7 expression is observed in st6gal1‐knockdown cells and is associated with a drop in the number of reserve cells

**Figure 5:**
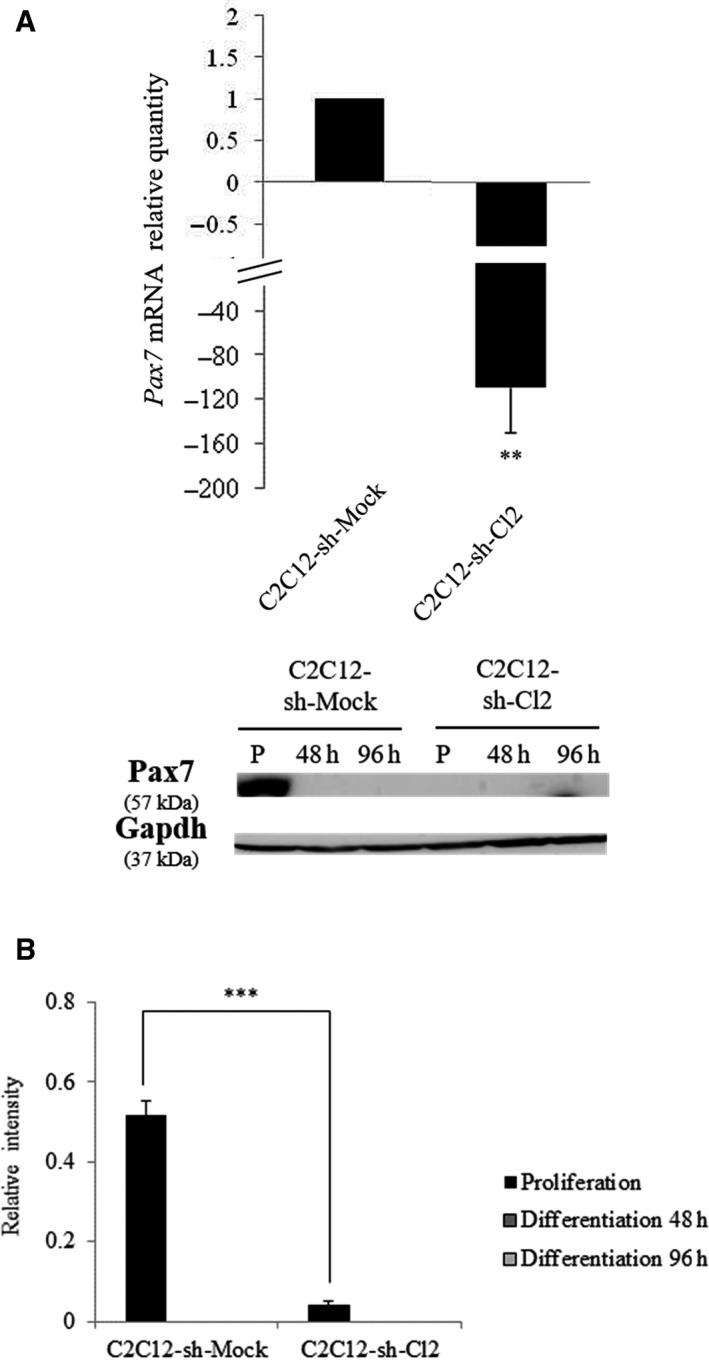
Knockdown of *st6gal1* reduced *Pax7* expression. (A) Expression of *Pax7* in C2C12‐sh‐Mock and C2C12‐sh‐Cl2 in proliferation. Fold changes are expressed relative to C2C12‐sh‐Mock cells. Data are expressed as relative transcript amounts corresponding to the ratio of *C*_t_ values for C2C12‐sh‐Cl2 cells and *C*_t_ values for C2C12‐sh‐Mock cells and normalized against the expression of *gapdh* transcript. Experiments have been done three times (*n* = 3). **P < 0.01; ***P < 0.001. (B) Relative Pax7 amounts determined by western blot using total cell proteins isolated from C2C12‐sh‐Mock and C2C12‐sh‐Cl2 in proliferation (P) and differentiation at 48 and 96 h. Protein levels were determined in comparison with gapdh loading control. Experiments have been done three times (*n* = 3). Means ± SEMs are shown (two‐tailed *t*‐test, with a significant level of **P < 0.01; ***P < 0.001).

**Figure 6:**
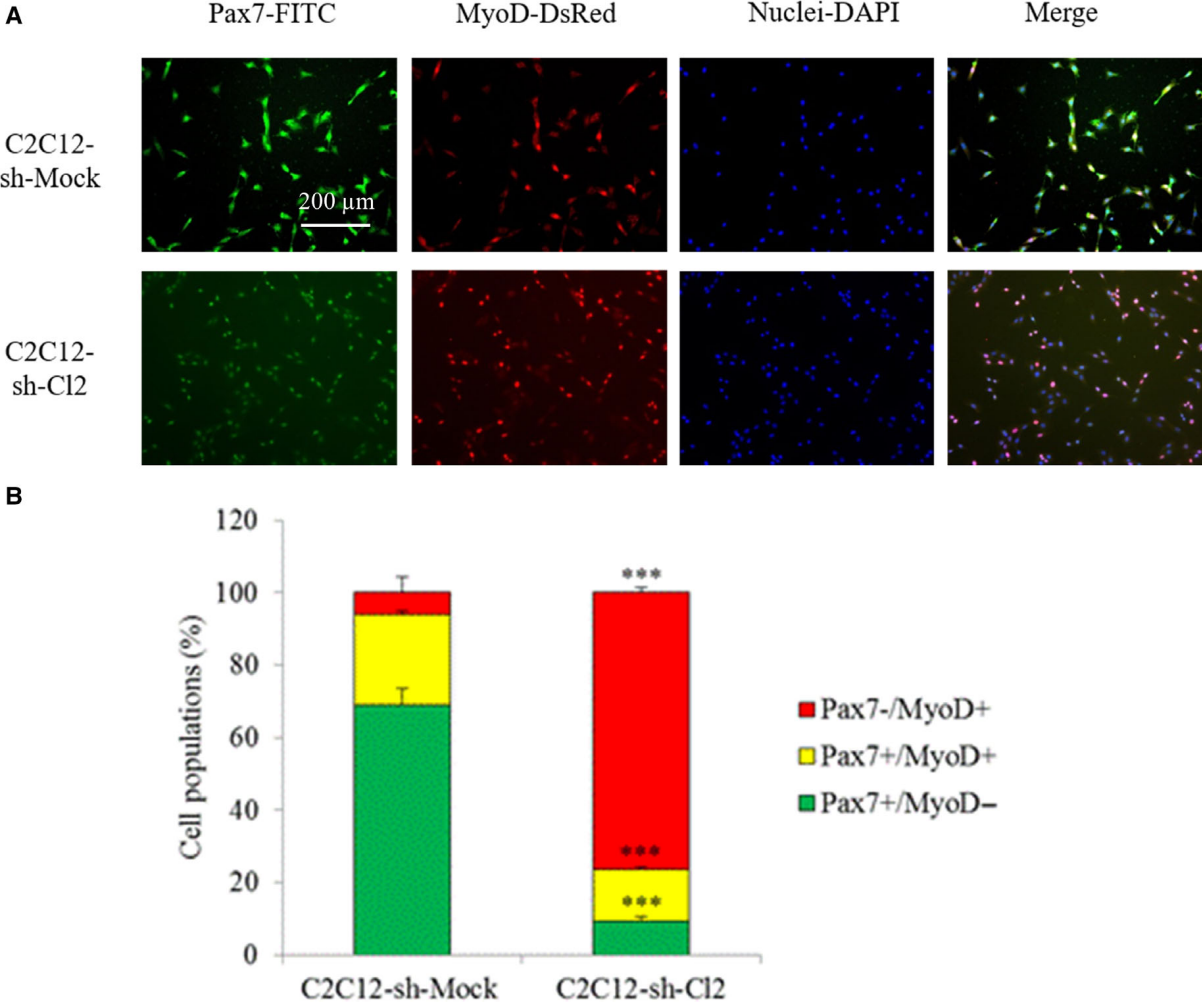
Effect of *st6gal1* knockdown on cell fate. (A) Co‐immunostaining of Pax7 (green) and MyoD (red) in C2C12‐sh‐Mock and C2C12‐sh‐Cl2 cells during proliferation. The exposure times were identical for a same staining between the two cell types (1000 ms for Pax7‐FITC and 500 ms for MyoD‐DsRed); scale bar: 200 µm. (B) Percentages of Pax7^+^/MyoD^−^, Pax7^+^/MyoD^+^, and Pax7^−^/MyoD^+^ cell populations during proliferation. Percentages are the mean of counting on seven fields by condition. Experiments have been done three times (*n* = 3). Means ± SEMs are shown (two‐tailed *t*‐test, with a significant level of ***P < 0.001).

During C2C12 differentiation, a population of reserve cells is maintained in an undifferentiated state, due to the expression of *Pax7* 25. *Pax7* expression level dramatically drops by 130‐fold in C2C12‐sh‐Cl2 in comparison with C2C12‐sh‐Mock (Fig. 5A). These results were confirmed by western blot analysis of cells during proliferation and at 48 and 96 h of differentiation (Fig. 5B). Protein Pax7 was virtually absent in *st6gal1‐*knockdown cells, either proliferative or differentiated. To determine which cells are actually impacted by this sharp fall in Pax7 expression, a dual staining of Pax7 and MyoD was performed on cells during proliferation (Fig. 6A). About 70% of the C2C12‐sh‐Mock cells were Pax7^+^/MyoD^−^ (self‐renewing cells also called reserve cells), 25% were Pax7^+^/MyoD^+^ (proliferating cells), and 5% were Pax7^−^/MyoD^+^ cells (differentiating cells; Fig. 6B). On the contrary, 75% of the C2C12‐sh‐Cl2 cells were found in a differentiated state (Pax7^−^/MyoD^+^). The weak amount of Pax7^+^ cells accounts for the virtual absence of Pax7 signal in the western blot (Fig. 5B).

### The loss of α2,6 sialylation impairs activation of the Notch signaling pathway

The Notch signaling pathway controls the transcription of *Pax7* and allows satellite cell renewal 26. It contributes to the expression of the *Hes* and *Hey* genes, which in turn inhibit *MyoD* expression and thus cell differentiation 27.

**Figure 7:**
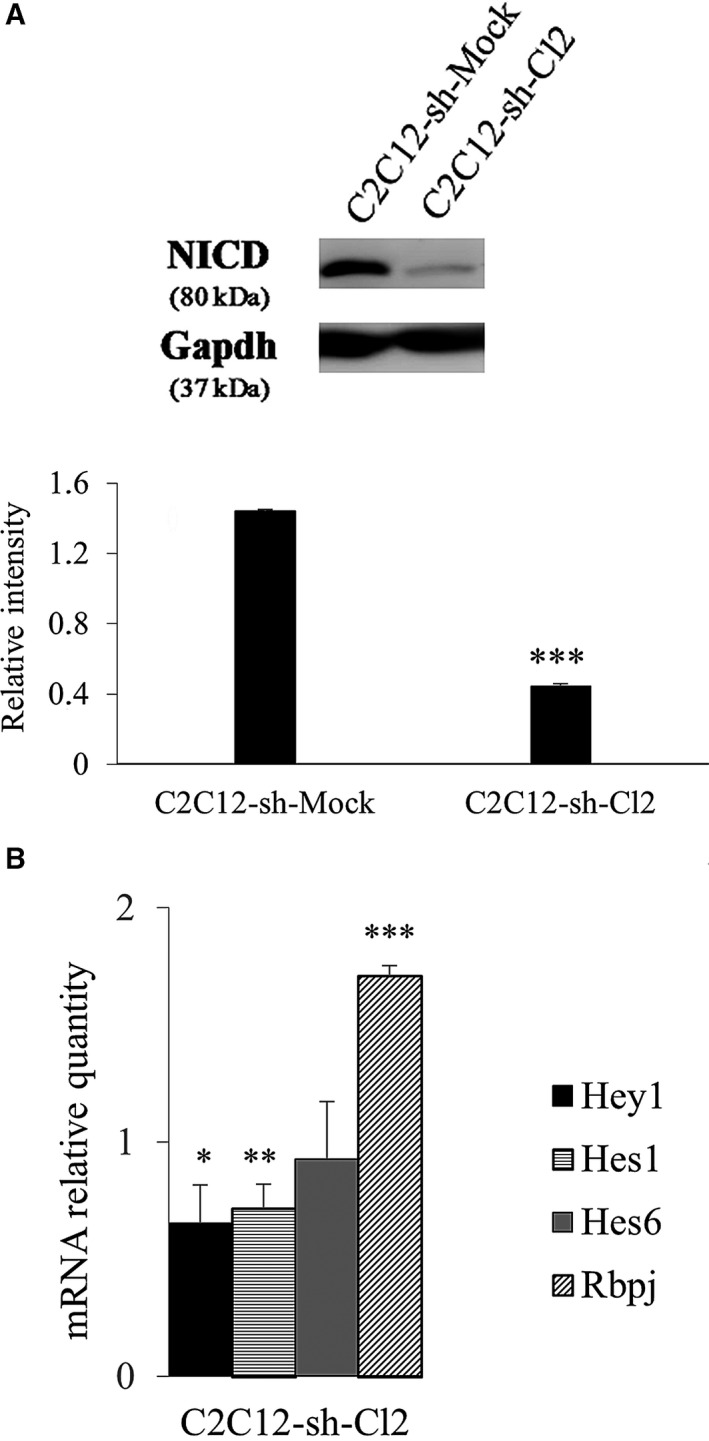
The expression of target genes of Notch, *Hey1* and *Hes1*, decreased in *st6gal1*‐knockdown cells. (A) Relative amounts of NICD (the active form of Notch which binds to Rbpj in order to transcriptionally activate target genes as *Hes1* and *Hey1*) determined by western blot using total proteins from proliferating C2C12‐sh‐Mock and C2C12‐sh‐Cl2 cells. Protein levels were determined in comparison with gapdh loading control. This experiment has been performed three times (*n* = 3). (B) Expression of Notch target genes *Hey1* and *Hes1* (encoding transcriptional repressors regulating myogenesis), *Hes6* whose expression is not under the control of Notch signaling (encoding an independent protein of the Notch signaling pathway) and *Rbpj* (encoding a transcription factor) in C2C12‐sh‐Mock and C2C12‐sh‐Cl2 cells in proliferation. Fold changes are expressed relative to C2C12‐sh‐Mock cells. Data are expressed as relative transcript amounts corresponding to the ratio of *C*_t_ values for C2C12‐sh‐Cl2 cells and *C*_t_ values for C2C12‐sh‐Mock cells and normalized against the expression of *gapdh* transcript. Statistical analyses of gene expression were performed by comparison with the C2C12‐sh‐Mock (which was set as 1) for three independent experiments (*n* = 3). Means ± SEMs are shown (two‐tailed *t*‐test, with a significant level of *P < 0.05; **P < 0.01; ***P < 0.001).

To know whether, and to which extent, the Notch signaling pathway is modified in *st6gal1*‐knockdown cells, the amount of NICD (the cleaved form of the Notch receptor) was evaluated by western blot analysis on C2C12‐sh‐Mock and C2C12‐sh‐Cl2 cells during proliferation (Fig. 7A). We observed a decrease of about 70% of NICD in C2C12‐sh‐Cl2 compared with C2C12‐sh‐Mock. Consequently, the weak activation of this pathway in *st6gal1*‐knockdown cells could imply their earlier entry into differentiation. To confirm the decrease in Notch signaling, we studied the expression of two genes under the control of Notch: *Hey1* and *Hes1* encoding myogenic differentiation repressors. *Hes6* is considered here as a control because its expression is independent of the Notch pathway 28. *Rbpj* is a transcription factor that, after its binding to NICD, activates the transcription of target genes such as *Hes1* and *Hey1* 18. We have performed a quantitative RT‐PCR on C2C12‐sh‐Mock and C2C12‐sh‐Cl2 cells in proliferation (Fig. 7B). In C2C12‐sh‐Cl2 cells, we observed a decrease in expression of *Hey1* and *Hes1* by 35% and 30%, respectively, and the expression of *Hes6* did not vary. *Rbpj* was also expressed in clone 2. Altogether, these results confirm the decrease in the Notch activation pathway, the decrease in *Pax7* expression and the earlier differentiation of *st6gal1*‐knockdown clone.

## Discussion

Sialic acids play important roles in the stabilization of macromolecules and membranes, as well as in modulating interactions of the cells with their environment 13. The roles of sialic acids in development are also documented. Unlike other tissues, the role of sialylation in muscle or during muscle development is currently poorly documented. Nevertheless, Suzuki and coworkers showed that, during myogenesis, the polysialylation of *N*‐glycans attached to neural cell adhesion molecule prevents myoblast fusion 29, but nothing has been reported about the α2,6 sialylation. We have reported in previous studies that changes in the expression levels of glycosylation‐related genes occurred during myogenic differentiation of C2C12 cells and murine satellite cells 10, 11. Among these genes, we have observed a deregulation of genes implicated in the Notch signaling pathway. In a first time, researches were focused on *Pofut1* and protein *O*‐glucosyltransferase 1, which respectively catalyze the *O*‐fucosylation and *O*‐glucosylation of EGF‐like domains, necessary for Notch activation during C2C12 cell proliferation 17, 30. A study about the implication of α2,6 sialylation during myoblast differentiation has not been studied yet.

We chose to knockdown *st6gal1* in C2C12 cells since among the two genes encoding for α2,6 sialyltransferases, it is the only one to be expressed in C2C12 cells. We have excluded the six *st6galNAc* genes whose products ensure α2,6 sialylation on *N*‐acetylgalactosamine residues. To get further information about the role of α2,6 sialylation in muscle development, we knocked down *st6gal1* in C2C12 cells. We found that the proliferation of *st6gal1*‐knockdown cells was slowed down, due to a generation time twice as long as control cells; however, these cells presented an increased fusion index (80% against 60% for control cells at 240 h of differentiation).

To improve our knowledge about the involvement of α2,6 sialylation during C2C12 cell differentiation and to determine which glycan structures are modified by the knockdown of *st6gal1*, we have in a first time evaluated the α2,3 and α2,6 linkages of sialic acids in mock and *st6gal1*‐knockdown cells. These observations were done during proliferation and differentiation conditions in order to appreciate the evolution of sialic acid amounts during myogenesis. The knockdown of *st6gal1* induced a significant decrease in α2,6‐linked sialic acids borne by *N*‐glycans on high molecular weight glycoproteins (> 55 kDa).

Mass spectrometric analyses revealed no modification of the proportions of sialylated *N*‐glycans between Mock and clone cells. It suggests that the *st6gal1* knockdown does not modify significatively the proportion of sialylated *N*‐glycans. After the myoblast differentiation, the *N*‐glycosylation profile of myotubes was completely modified; myotubes presented much more sialylated glycans, while the oligomannosylated glycans dramatically dropped. The five sialylated and nonfucosylated glycans are less represented in the C2C12‐sh‐Cl2 reserve cells compared to those in proliferation. We observed a similar trend for Mock cells, excepted for the tetrasialylated glycan (*m*/*z*: 3966), which increased in the reserve cells compared to the proliferative condition. As their amounts decreased in reserve cells, these sialylated and nonfucosylated structures would not be involved in the maintenance of these cells.

Despite the loss of sialylation, the percentage of fucosylated structures did not change between cell types during proliferation. However, sialylated and bifucosylated glycans (*m*/*z*: 2778, 2822, 3023, 3228) overall slightly decreased by 1.2‐fold in myotubes of *st6gal1*‐knockdown cells, compared with myotubes of Mock cells.

Biantennary bisialylated (*m*/*z*: 2792), triantennary bisialylated (*m*/*z*: 3242), and biantennary bifucosylated monosialylated (*m*/*z*: 2778, 2822) glycans were systematically found in a larger amount in myotubes in comparison with proliferating cells and reserve cells. These sialylated species could promote the myocyte fusion and the formation of myotubes. In contrast, the fucosylated and nonsialylated glycan (*m*/*z*: 2693) identified in this study might be an inhibitor of myogenic fusion since it has not been found in myotubes of the two cell types whereas it was always present in proliferating and reserve cells.

We have highlighted a drop in proliferative capacity linked to a decrease in α2,6 sialylation. To explain this phenomenon, we have analyzed the transcriptional and protein levels of *Pax7*. Pax7^+^ cells can be activated to initiate the myogenic differentiation program or stay in a quiescence state as reserve cells 8, 9. We showed that the expression level of *Pax7*, which is required for cell proliferation of C2C12 myoblasts 31, is very low in *st6gal1*‐knockdown cells; this could explain the more important fusion index of these cells as also observed by Der Vartanian *et al*. when *Pax 7* expression was repressed in C2C12 cells 17.

*Pax7* depends on the Notch pathway, and Notch signaling targets genes that control the behavior of satellite cells 32. The lower amount of NICD, corresponding to the cleaved form of Notch, observed when α2,6 sialylation decreased, reflects a downregulation of Notch signaling. Thus, the loss of α2,6 sialylation induced a decrease in Notch pathway activation, which was confirmed by the decrease in expression of the Notch target genes *Hes1* and *Hey1* in *st6gal1*‐knockdown cells. *Hes1* and *Hey1* encode repressors of myogenic differentiation, which inactivate the expression of MRF as MyoD. Notch pathway activation controls the ratio Pax7/MyoD in favor of Pax7. When α2,6 sialylation decreases, the weak Notch signaling induces a strong decrease in *Pax7* expression, and so, the differentiation of cells is promoted. We hypothesize that the decrease in sialylation may affect receptors that would be involved in the Notch signaling pathway (or other pathways) that results in reduced expression level of Pax7.

In conclusion, we have observed that the *st6gal1*‐knockdown promotes C2C12 cell differentiation. This may be due to a decrease in Notch signaling and finally to a decrease in *Pax7* expression. Moreover, the lower expression of *st6gal1* induces a lower proliferation of *st6gal1*‐knockdown cells in relation with a poor expression of *Pax7*. Three types of *N*‐glycans seem to be required for myogenic differentiation: biantennary bisialylated, triantennary bisialylated, and biantennary monosialylated bifucosylated. The early fusion of *st6gal1‐*knockdown cells and its high level explain the drop in self‐renewing cells Pax7^+^/MyoD^‐^. This study showed the consequences of α2,6 knockdown on Notch signaling. However, we cannot exclude that other receptors could be impacted by the decrease in α2,6 sialylation. In conclusion, α2,6 sialylation (a) seems mandatory for *Pax7* activation and so for cellular proliferation; (b) contributes to keep up the pool of reserve cells; and (c) limits myoblastic fusion.

## Conflict of interest

The authors declare no conflict of interest.

## Author contributions

JMP and AM conceived and designed the project. CV, AB, FC, and YG acquired the data. CV, FC, YG, and JMP drafted the manuscript. AM critically revised the manuscript.

## Supporting information


**Fig. S1.** Validation of the *st6gal1* knockdown phenotype with another clone.

**Fig. S2.** Treatment by PNGase reduced SNA and MAA binding.

**Fig. S3.** MS profiles of sialylated and nonsialylated *N*‐glycans of C2C12‐sh‐Mock and C2C12‐sh‐Cl2 cells.

**Fig. S4.** Structures of *N*‐glycans identified by MS/MS spectrometry.

**Fig. S5.** Distribution relative of *N*‐glycans in C2C12‐sh‐Mock and C2C12‐sh‐Cl2 cells.

## References

[1] Yoshida N , Yoshida S , Koishi K , Masuda K and Nabeshima Y (1998) Cell heterogeneity upon myogenic differentiation: down‐regulation of MyoD and Myf‐5 generates 'reserve cells'. J Cell Sci 111, 769–779. 9472005 10.1242/jcs.111.6.769

[2] Olguin HC and Pisconti A (2012) Making the tempo of myogenesis: Pax7 and the regulation of muscle stem cell fate decisions. J Cell Mol Med 16, 1013–1025. 21615681 10.1111/j.1582-4934.2011.01348.x PMC4365881

[3] Choi J , Costa ML , Mermelstein CS , Chagas C , Holtzer S and Holtzer H (1990) MyoD converts primary dermal fibroblasts, chondroblasts, smooth muscle, and retinal pigmented epithelial cells into striated mononucleated myoblasts and multinucleated myotubes. Proc Natl Acad Sci USA 87, 7988–7992. 2172969 10.1073/pnas.87.20.7988 PMC54877

[4] Weintraub H , Tapscott SJ , Davis RL , Thayer MJ , Adam MA , Lasser AB and Miller AD (1991) Activation of muscle‐specific genes in pigment, nerve, fat, liver and fibroblast cell lines by forced expression of MyoD. Proc Natl Acad Sci USA 86, 5434–5438. 10.1073/pnas.86.14.5434 PMC297637 2748593

[5] Parker MH , Seale P and Rudnicki MA (2003) Looking back to the embryo: defining transcriptional networks in adult myogenesis. Nat Rev Genet 4, 497–507. 12838342 10.1038/nrg1109

[6] Zanou N and Gailly P (2013) Skeletal muscle hypertrophy and regeneration: interplay between the myogenic regulatory factors (MRFs) and insulin‐like growth factors (IGFs) pathways. Cell Mol Life Sci 70, 4117–4130. 23552962 10.1007/s00018-013-1330-4 PMC11113627

[7] Moncaut N , Rigby PW and Carvajal JJ (2013) Dial M(RF) for myogenesis. FEBS J 280, 3980–3990. 23751110 10.1111/febs.12379

[8] Relaix F , Montarras D , Zaffran S , Gayraud‐Morel B , Rocancourt D , Tajbakhsh S , Mansouri A , Cumano A and Buckingham M (2006) Pax3 and Pax7 have distinct and overlapping functions in adult muscle progenitor cells. J Cell Biol 172, 91–102. 16380438 10.1083/jcb.200508044 PMC2063537

[9] Kuang S , Kuroda K , Le Grand F and Rudnicki MA (2007) Asymmetric self‐renewal and commitment of satellite stem cells in muscle. Cell 125, 999–1010. 10.1016/j.cell.2007.03.044 PMC2718740 17540178

[10] Janot M , Audfray A , Loriol C , Germot A , Maftah A and Dupuy F (2009) Glycogenome expression dynamics during mouse C2C12 myoblast differentiation suggests a sequential reorganization of membrane glycoconjugates. BMC Genom 10, 483. 10.1186/1471-2164-10-483 PMC2772862 19843320

[11] Grassot V , Da Silva A , Saliba J , Maftah A , Dupuy F and Petit JM (2014) Highlights of glycosylation and adhesion related genes involved in myogenesis. BMC Genom 15, 621. 10.1186/1471-2164-15-621 PMC4223822 25051993

[12] Varki NM and Varki A (2007) Diversity in cell surface sialic acid presentations: implications for biology and disease. Lab Invest 87, 851–857. 17632542 10.1038/labinvest.3700656 PMC7100186

[13] Schauer R (2009) Sialic acids as regulators of molecular and cellular interactions. Curr Opin Struct Biol 19, 507–514. 19699080 10.1016/j.sbi.2009.06.003 PMC7127376

[14] Varki A and Gagneux P (2012) Multifarious roles of sialic acids in immunity. Ann NY Acad Sci 1253, 16–36. 22524423 10.1111/j.1749-6632.2012.06517.x PMC3357316

[15] Harduin‐Lepers A , Vallejo‐Ruiz V , Krzewinski‐Recchi MA , Samyn‐Petit B , Julien S and Delannoy P (2001) The human sialyltransferase family. Biochimie 83, 727–737. 11530204 10.1016/s0300-9084(01)01301-3

[16] Lehoux S , Groux‐Degroote S , Cazet A , Dhaenens CM , Maurage CA , Caillet‐Boudin ML , Delannoy P and Krzewinski‐Recchi MA (2010) Transcriptional regulation of the human ST6GAL2 gene in cerebral cortex and neuronal cells. Glycoconj J 27, 99–114. 19768537 10.1007/s10719-009-9260-y

[17] Der Vartanian A , Audfray A , Al Jaam B , Janot M , Legardinier S , Maftah A and Germot A (2015) Protein O‐fucosyltransferase 1 expression impacts myogenic C2C12 cell commitment via the Notch signaling pathway. Mol Cell Biol 35, 391–405. 25384974 10.1128/MCB.00890-14 PMC4272425

[18] Mayeuf A and Relaix F (2011) Notch pathway: from development to regeneration of skeletal muscle. Med Sci 27, 521–526. 10.1051/medsci/2011275018 21609674

[19] Kopan R , Nye JS and Weintraub H (1994) The intracellular domain of mouse Notch: a constitutively activated repressor of myogenesis directed at the basic helix–loop–helix region of MyoD. Development 120, 2385–2396. 7956819 10.1242/dev.120.9.2385

[20] Kitzmann M , Carnac G , Vandromme M , Primig M , Lamb NJ and Fernandez A (1998) The muscle regulatory factors MyoD and Myf‐5 undergo distinct cell cycle‐specific expression in muscle cells. J Cell Biol 142, 1147–1459. 10.1083/jcb.142.6.1447 PMC2141770 9744876

[21] Velica T and Bunce CM (2011) A quick, simple and unbiased method to quantify C2C12 myogenic differentiation. Muscle Nerve 44, 366–370. 21996796 10.1002/mus.22056

[22] Le Cam L , Linares LK , Paul C , Julien E , Lacroix M , Hatchi E , Triboulet R , Bossis G , Shmueli A , Rodriguez MS *et al* (2006) E4F1 is an atypical ubiquitin ligase that modulates p53 effector functions independently of degradation. Cell 127, 775–788. 17110336 10.1016/j.cell.2006.09.031

[23] Ciucanu I and Kerek F (1984) Rapid and simultaneous methylation of fatty and hydroxy fatty acids for gas‐liquid chromatographic analysis. J Chromatogr A 284, 179–185.

[24] Livak KJ and Schmittgen TD (2001) Analysis of relative gene expression data using real‐time quantitative PCR and the 2(‐Delta DeltaC(T)) method. Methods 25, 402–408. 11846609 10.1006/meth.2001.1262

[25] Olguin HC and Olwin BB (2004) Pax‐7 up‐regulation inhibits myogenesis and cell cycle progression in satellite cells: a potential mechanism for self‐renewal. Dev Biol 275, 375–388. 15501225 10.1016/j.ydbio.2004.08.015 PMC3322464

[26] Wen Y , Bi P , Liu W , Asakura A , Keller C and Kuang S (2012) Constitutive Notch activation upregulates Pax7 and promotes the self‐renewal of skeletal muscle satellite cells. Mol Cell Biol 32, 2300–2311. 22493066 10.1128/MCB.06753-11 PMC3372272

[27] Kuroda K , Tani S , Tamura K , Minoguchi S , Kurooka H and Honjo T (1999) Delta‐induced Notch signaling mediated by RBP‐J inhibits MyoD expression and myogenesis. J Biol Chem 274, 7238–7244. 10066785 10.1074/jbc.274.11.7238

[28] Cossins J , Vernon AE , Zhang Y , Philpott A and Jones PH (2002) Hes6 regulates myogenic differentiation. Development 129, 2195–2207. 11959828 10.1242/dev.129.9.2195

[29] Suzuki M , Angata K , Nakayama J and Fukuda M (2003) Polysialic acid and mucin type‐O‐glycans on the neural cell adhesion molecule differentially regulate myoblast fusion. J Biol Chem 278, 49459–49468. 13679364 10.1074/jbc.M308316200

[30] Pélisse M , Der Vartanian A , Germot A and Maftah A (2018) Protein O‐glucosyltransferase 1 expression influences formation of differentiated myotubes in C2C12 cell line. DNA Cell Biol 37, 359–372. 29634421 10.1089/dna.2017.4052

[31] Collins CA , Gnocchi VF , White RB , Boldrin L , Perez‐Ruiz A , Relaix F , Morgan JE and Zammit PS (2009) Integrated functions of Pax3 and Pax7 in the regulation, cell size and myogenic differentiation. PLoS ONE 4, e4475. 19221588 10.1371/journal.pone.0004475 PMC2637421

[32] Mourikis P , Sambasivan R , Castel D , Rocheteau P , Bizzarro V and Tajbakhshs S (2012) A critical requirement for notch signaling in maintenance of the quiescent skeletal stem cell state. Stem Cells 30, 243–252. 22069237 10.1002/stem.775

