# Supplementary material for: Involvement of ST6Gal I‐mediated α2,6 sialylation in myoblast proliferation and differentiation
Source: FEBS Open Bio. 2019 Dec 10;10(1):56–69. doi: 10.1002/2211-5463.12745 (PMC6943236; doi:10.1002/2211-5463.12745)
Supplement: Supplementary file 1 — Fig. S1. Validation of the st6gal1 knockdown phenotype with another clone. [file FEB4-10-56-s001.pdf]

A.

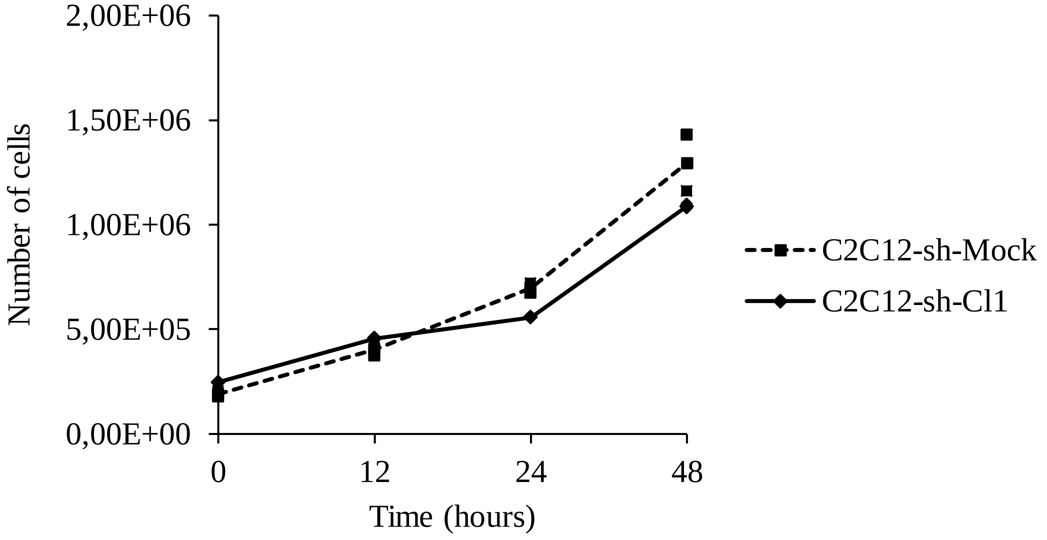

B.

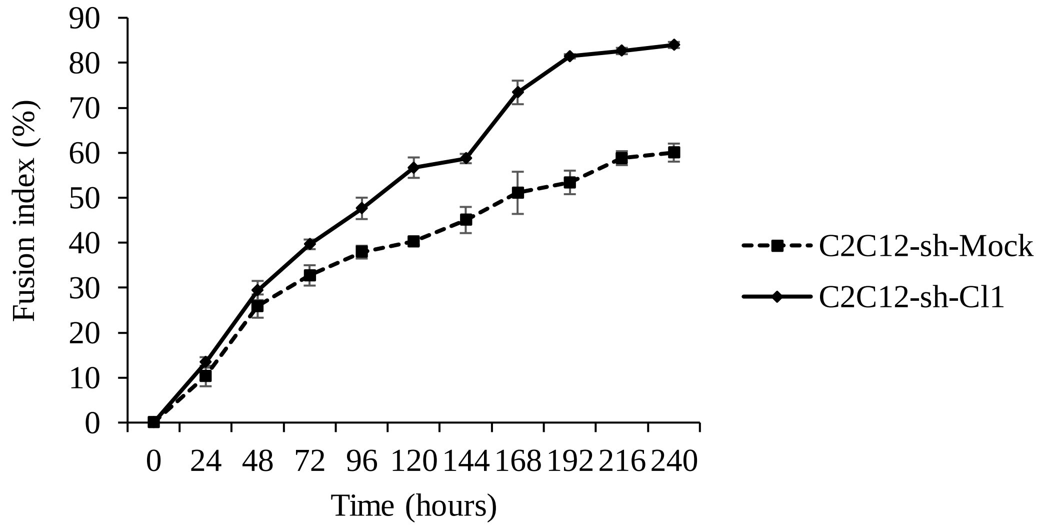

Figure S1

Validation of the *st6gal1* knockdown phenotype with another clone. (A) Proliferation rate of C2C12-sh-Mock cells (dotted line) and C2C12-sh-Cl1 cells (solid line). Two different sets of measures are shown (n=2), and the average curve is represented. **(B)** Differentiation rate of C2C12-sh-Mock (dotted line) and C2C12-sh-Cl1 (solid line). Cell fusion was measured at various times by Jenner-Giemsa staining and expressed as fusion index (%). Vertical bares denote SEM for ten observation fields of the same experience.
