# Supplementary figures and images for: Involvement of ST6Gal I‐mediated α2,6 sialylation in myoblast proliferation and differentiation
Source: FEBS Open Bio. 2019 Dec 10;10(1):56–69. doi: 10.1002/2211-5463.12745 (PMC6943236; doi:10.1002/2211-5463.12745)

A.

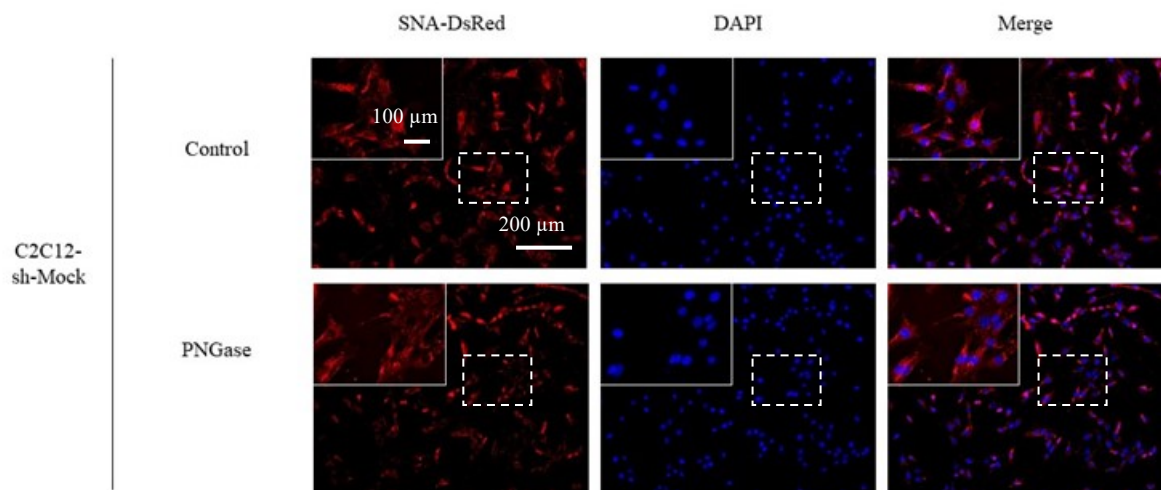

B.

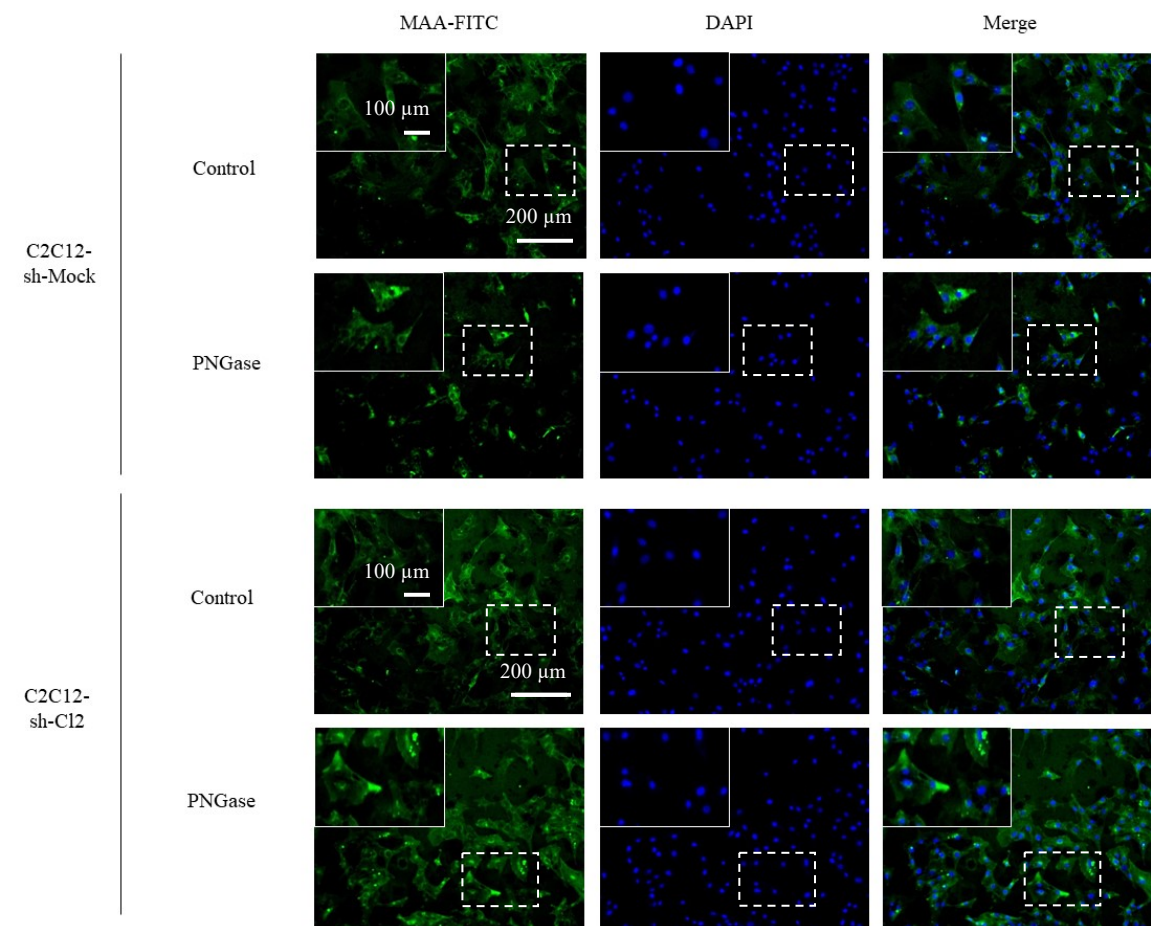

Figure S2

Supplement: Supplementary file 2 — Fig. S2. Treatment by PNGase reduced SNA and MAA binding. [file FEB4-10-56-s002.pdf]
