# Supplementary material for: Involvement of ST6Gal I‐mediated α2,6 sialylation in myoblast proliferation and differentiation
Source: FEBS Open Bio. 2019 Dec 10;10(1):56–69. doi: 10.1002/2211-5463.12745 (PMC6943236; doi:10.1002/2211-5463.12745)
Supplement: Supplementary file 3 — Fig. S3. MS profiles of sialylated and nonsialylated N‐glycans of C2C12‐sh‐Mock and C2C12‐sh‐Cl2 cells. [file FEB4-10-56-s003.pdf]

A. Proliferation

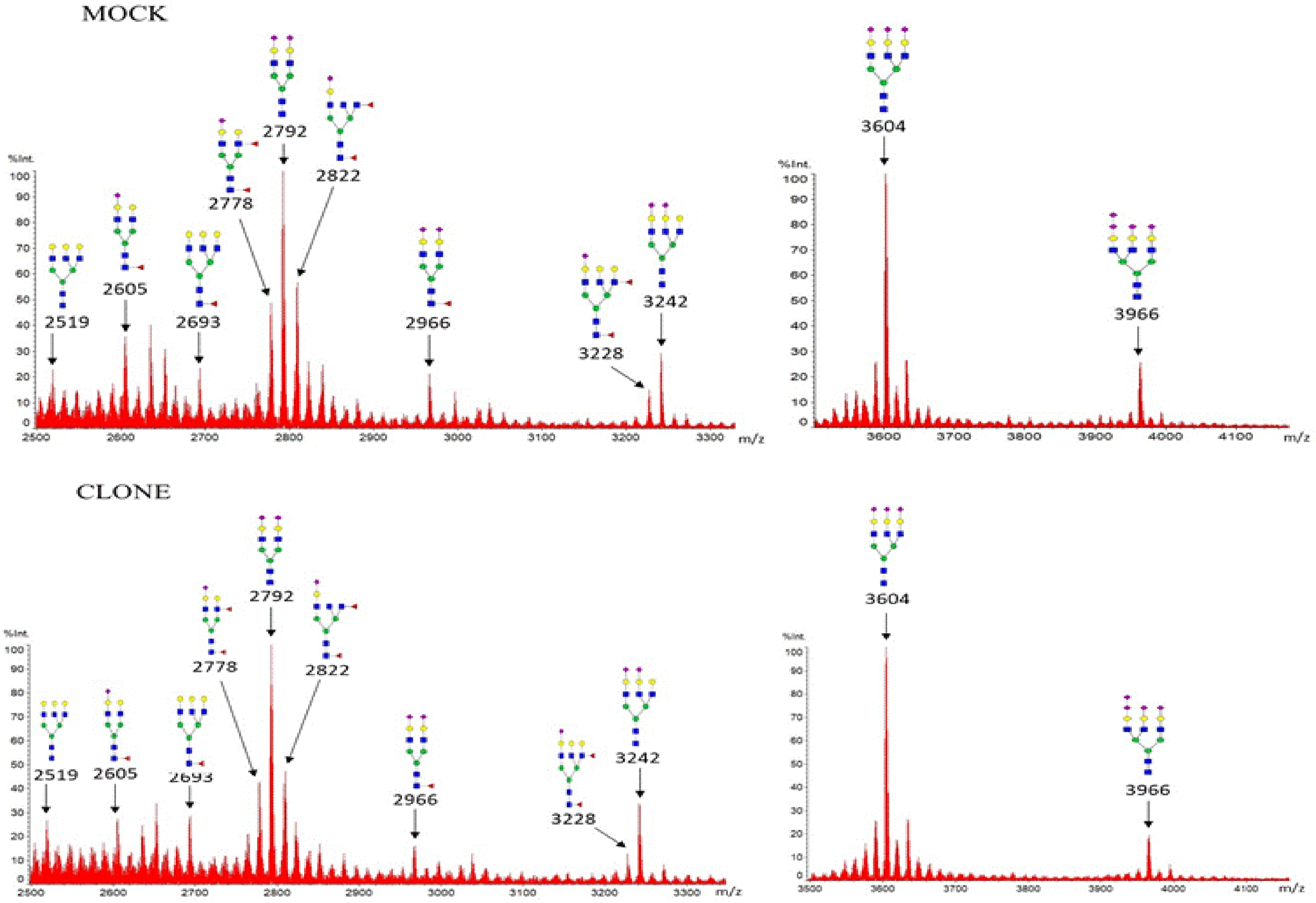

B. Differentiation 120 h MT

MOCK

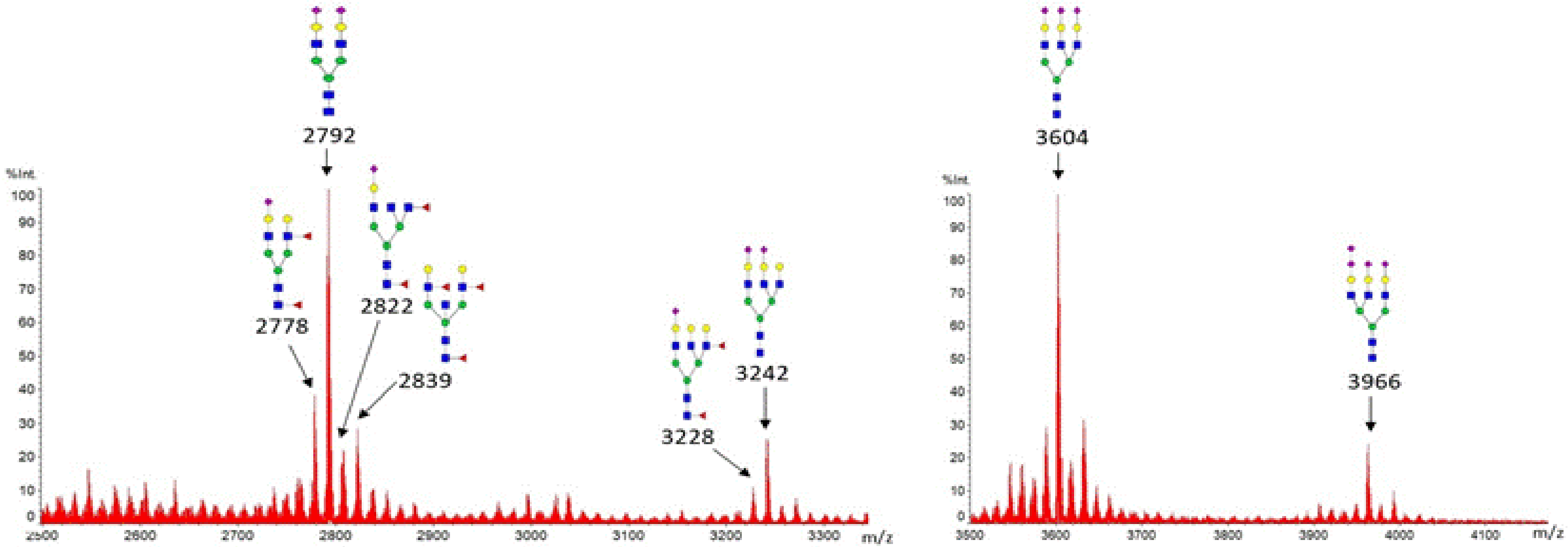

CLONE

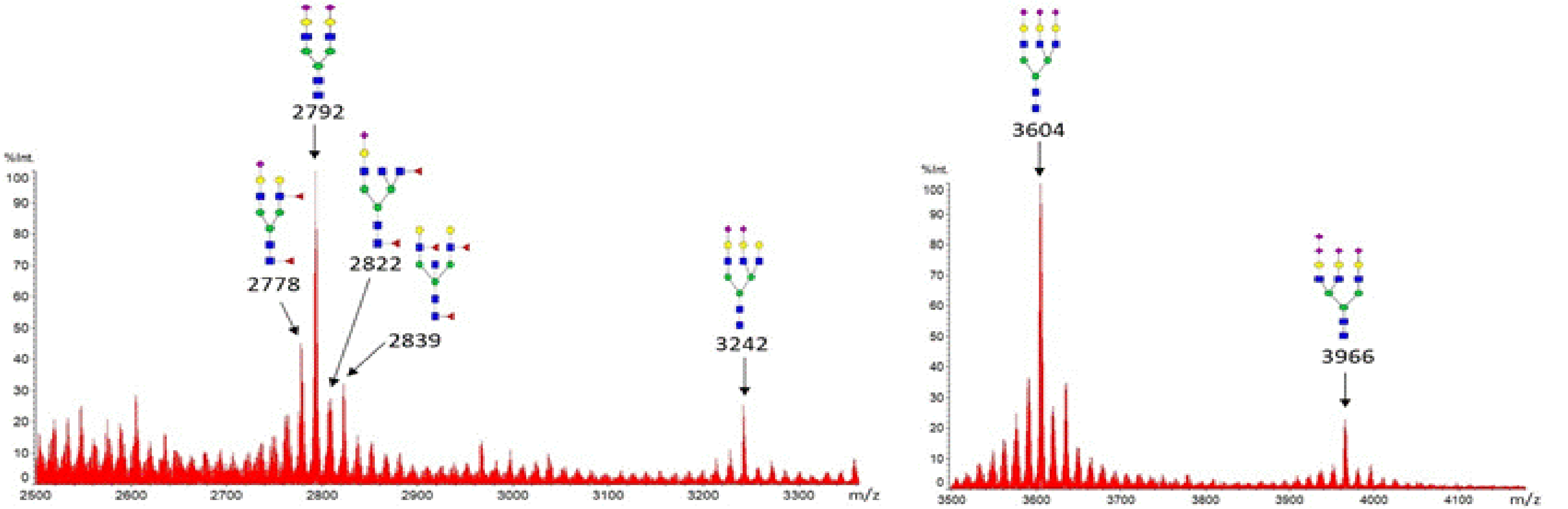

C. Differentiation 120 h RC+MB

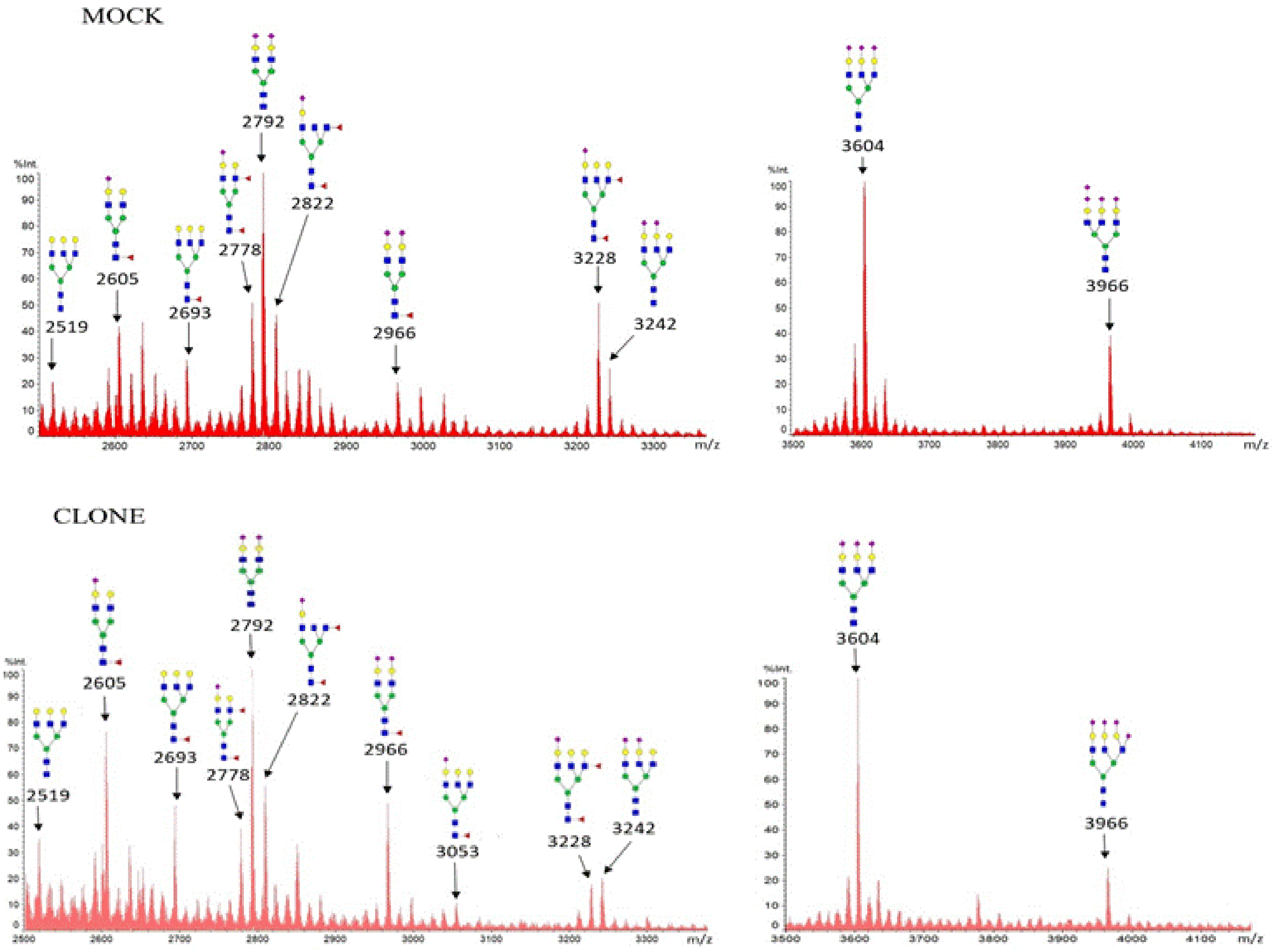

MS profiles of sialylated and non-sialylated *N*-glycans of C2C12-sh-Mock and C2C12-sh-Cl2 cells. The C2C12-sh-Mock (upper profile) and C2C12-sh-Cl2 (low profile)**:** proliferating cells (A); myotubes at 120 hours of differentiation (B) and reserve cells and myoblasts at 120 hours of differentiation (C). The spectra show the different peaks corresponding to the identified glycans from 2500 to 3300 m/z (left spectrum) and from 3500 to 4200 m/z (right spectrum). For each spectrum, the intensity for the maximal peak was fixed at 100% and other intensities were calculated relative this value. Mass spectrometry analysis have been done one time (n=1). Cells of three independent cultures have been pooled before *N*-glycans extraction and analyse.
