# Supplementary material for: Involvement of ST6Gal I‐mediated α2,6 sialylation in myoblast proliferation and differentiation
Source: FEBS Open Bio. 2019 Dec 10;10(1):56–69. doi: 10.1002/2211-5463.12745 (PMC6943236; doi:10.1002/2211-5463.12745)
Supplement: Supplementary file 4 — Fig. S4. Structures of N‐glycans identified by MS/MS spectrometry. [file FEB4-10-56-s004.pdf]

		N-glycan structure	Mass (m/z)	C2C12-sh-Mock			C2C12-sh-Cl2		
				Prolife-ration	Differentiation		Prolife-ration	Differentiation	
					120 h MT	120 h RC+MB		120 h MT	120 h RC+MB
Oligomannosylated glycans		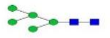	1579	242.81	29.61	167.45	122.99	31.13	235.65
		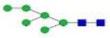	1783	156.52	28.87	176.23	151.42	39.41	403.92
		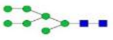	1988	94.02	9.29	83.7	70.74	11.3	166.76
		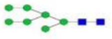	2192	91.95	10.91	103.91	76.65	14.09	202.85
		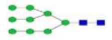	2395	87.63	9.67	104.44	51.92	0	184.71
Complex glycans	Neu-tral	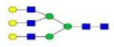	2519	75.76	0	65.33	55.6	0	90.84
	Fucosylated	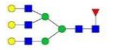	2693	76.43	0	82.69	64.64	0	111
		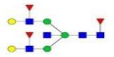	2839	83.39	0	79.01	0	0	0
	Sialylated	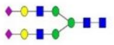	2792	336.4	610.85	278.92	215.85	303.82	249.27
		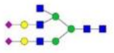	3037	0	55.44	0	27.59	0	0
		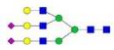	3242	100.45	169.24	69.61	71.57	74.16	51.52
		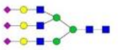	3604	494.64	331.88	377.62	345.26	337.53	237.58
		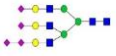	3966	127.83	82.05	151.39	73.98	72.61	68.86
	Fucosylated and sialylated	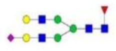	2605	122.38	81.2	122.77	65.15	83.33	189.2
		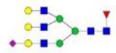	3053	0	0	23.88	0	0	28.86
		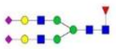	2966	62.09	40.63	59.75	36.86	45.15	115.85
		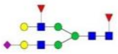	2778	168.63	230	146.32	95.73	142.85	92.14
		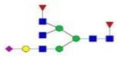	2822	85.47	167.58	64.68	57.09	98.99	0
		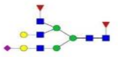	3023	0	50.52	44.73	0	0	0
		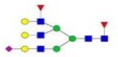	3228	49.62	68.8	136.62	27.94	37.31	46.75
		**Total**		2456.02	1976.54	2339.05	1610.98	1291.68	2475.76

Figure S4

Structures of *N*-glycans identified by MS/MS spectrometry. The identified glycans are classified according to their structure (oligomannose or complex) and their glycosylation state (fucosylated and/or sialylated). Each peak area is specified for C2C12-sh-Mock and C2C12-sh-Cl2 cells in proliferation (P) and in myotubes (MT) or in the mix of reserve cells (RC) and myoblasts (MB).
