# Supplementary material for: Involvement of ST6Gal I‐mediated α2,6 sialylation in myoblast proliferation and differentiation
Source: FEBS Open Bio. 2019 Dec 10;10(1):56–69. doi: 10.1002/2211-5463.12745 (PMC6943236; doi:10.1002/2211-5463.12745)
Supplement: Supplementary file 5 — Fig. S5. Distribution relative of N‐glycans in C2C12‐sh‐Mock and C2C12‐sh‐Cl2 cells. [file FEB4-10-56-s005.pdf]

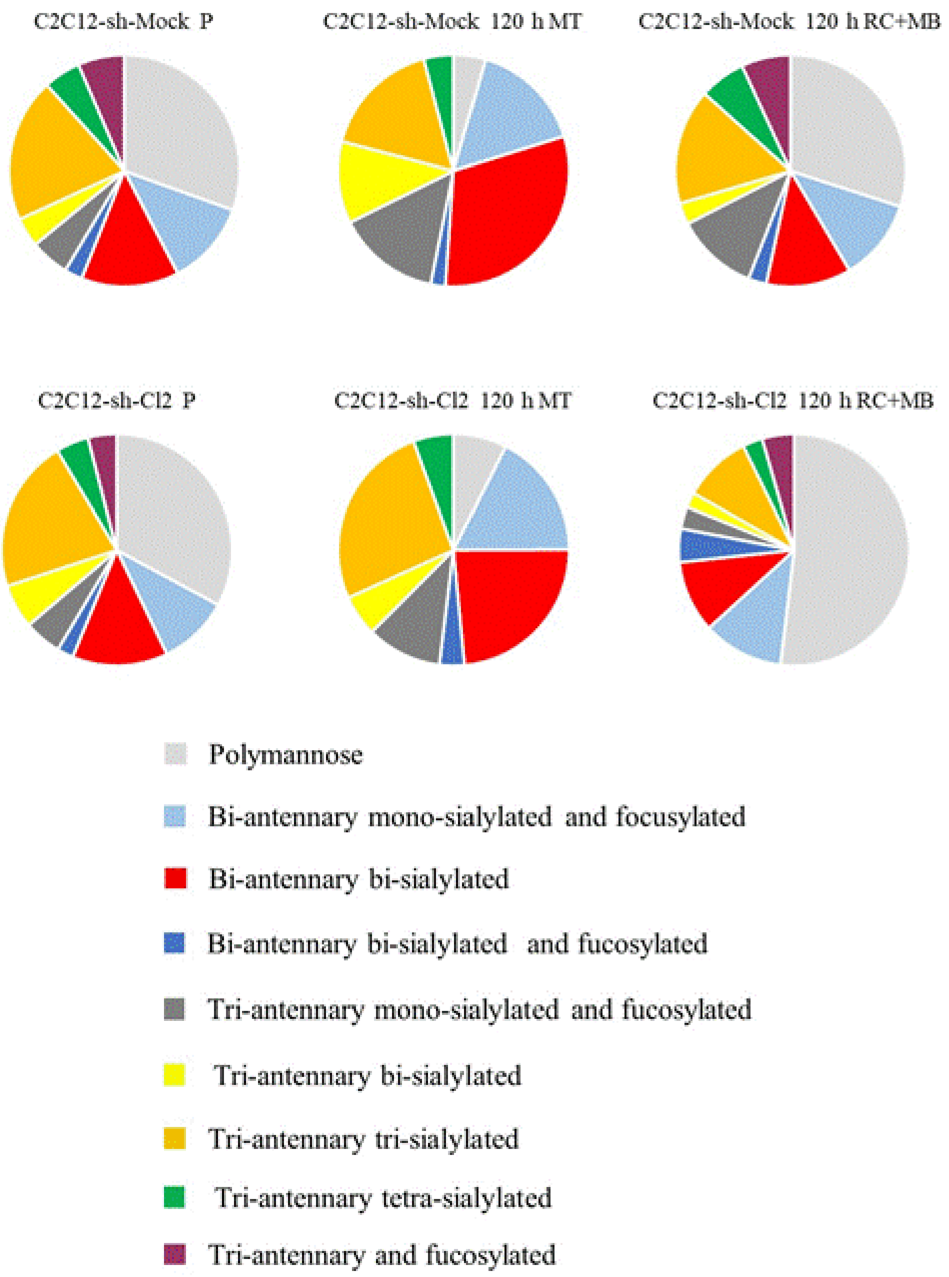

Figure S5

Distribution relative of *N*-glycans in C2C12-sh-Mock and C2C12-sh-Cl2 cells. Parts of each type of *N*-glycans identified by MS/MS spectrometry are given for proliferating myoblasts (P), and at 120 hours of differentiation for myotubes (MT), and the mix of reserve cells (RC) and myoblasts (MB) for C2C12-sh-Mock and C2C12-sh-Cl2 cells.
